# Lateral gate dynamics of the bacterial translocon during cotranslational membrane protein insertion

**DOI:** 10.1073/pnas.2100474118

**Published:** 2021-06-23

**Authors:** Evan Mercier, Xiaolin Wang, Manisankar Maiti, Wolfgang Wintermeyer, Marina V. Rodnina

**Affiliations:** ^a^Department of Physical Biochemistry, Max Planck Institute for Biophysical Chemistry, 37077 Göttingen, Germany

**Keywords:** single-molecule biophysics, YidC, membrane proteins, ribosome, translocon SecYEG

## Abstract

During synthesis of membrane proteins, transmembrane segments (TMs) of nascent proteins emerging from the ribosome are inserted into the central pore of the translocon (SecYEG in bacteria) and access the phospholipid bilayer through the open lateral gate formed of two helices of SecY. Here we use single-molecule fluorescence resonance energy transfer to monitor lateral-gate fluctuations in SecYEG embedded in nanodiscs containing native membrane phospholipids. We find the lateral gate to be highly dynamic, sampling the whole range of conformations between open and closed even in the absence of ligands, and we suggest a statistical model-free approach to evaluate the ensemble dynamics. Lateral gate fluctuations take place on both short (submillisecond) and long (subsecond) timescales. Ribosome binding and TM insertion do not halt fluctuations but tend to increase sampling of the open state. When YidC, a constituent of the holotranslocon, is bound to SecYEG, TM insertion facilitates substantial opening of the gate, which may aid in the folding of YidC-dependent polytopic membrane proteins. Mutations in lateral gate residues showing in vivo phenotypes change the range of favored states, underscoring the biological significance of lateral gate fluctuations. The results suggest how rapid fluctuations of the lateral gate contribute to the biogenesis of inner-membrane proteins.

## Significance

Membrane proteins are inserted into the phospholipid bilayer through a lateral gate in the translocon, SecYEG in bacteria, which is expected to be closed in the resting state. Here, we use single-molecule FRET to study the translocon dynamics on timescales ranging from submilliseconds to seconds. We show that the lateral gate is highly dynamic, fluctuating through a continuum of states from open to closed. The insertase YidC facilitates the insertion of transmembrane helices by shifting the fluctuations toward more open conformations. Spontaneous fluctuations allow the gate to rapidly release newly synthesized transmembrane segments into the phospholipid bilayer during ongoing translation. The results highlight the important role of rapid spontaneous fluctuations during the key step in the biogenesis of inner-membrane proteins.

The translocon is a transmembrane protein complex that allows newly synthesized proteins to be inserted into, and translocated across, the inner membrane of bacteria or the endoplasmic reticulum membrane of eukaryotes. Integral membrane proteins are targeted to the translocon as ribosome-nascent-chain complexes (RNCs) through the coordinated action of the signal recognition particle (SRP) and the SRP receptor (reviewed in refs. 1–3). Transmembrane segments (TMs) can then be integrated into the phospholipid bilayer cotranslationally after passing from the peptide exit tunnel of the ribosome into the adjacent pore of the translocon (reviewed in ref. 4). The TMs of nascent membrane proteins can interact with the translocon, adjacent TMs, or other membrane proteins, but the dynamics of these interactions have not been studied in detail (5–7).

The wealth of structural information available for the universally conserved translocon spans all domains of life. In bacteria, the core translocon is composed of three subunits: SecY, SecE, and SecG. The largest subunit, SecY, forms a channel with 10 TMs arranged around a central pore and a plug domain covering the periplasmic opening. Following successful targeting, an RNC binds to the cytosolic face of the translocon, allowing TMs of the nascent protein to pass from the peptide exit tunnel of the ribosome into the translocon pore. The translocon can open in two ways: 1) Movement of the plug domain permits translocation of proteins through the central conduit, while 2) opening of the lateral gate formed by TM2 and TM7 of SecY permits TMs to pass from the translocon pore into the phospholipid bilayer (8–10). In some instances, TMs have been observed in proximity to and/or interacting with the lateral gate after lipid integration, which could facilitate later folding events in the membrane (11, 12). The molecular details of what occurs at this stage are unclear, but the lateral gate presumably closes after TM insertion, in particular to help direct cytoplasmic or periplasmic loops to the correct side of the membrane. A number of mutations that map to the lateral gate of the translocon (13) confer various phenotypes, which include cold sensitivity and translocation of nascent peptides with mutated signal sequences, indicating an important role of the lateral gate in maintaining cellular homeostasis.

In bacteria, the SecYEG core translocon can function alone, in complex with the accessory factor YidC, or as the central component of a larger holotranslocon complex, which includes accessory factors YidC, SecDF, and YajC. In the cell, SecDF and YajC are found at substoichiometric levels relative to SecYEG and bring about the ATP-dependent translocation of proteins across the inner membrane (2, 14, 15). YidC, on the other hand, outnumbers the core translocon components and also functions as a stand-alone insertase for specific membrane proteins (16–18). The simpler SecYEG and SecYEG-YidC complexes are the best-studied, and both are functional for insertion of membrane proteins (19–22). YidC interacts with the lateral gate of SecYEG (23) and contacts TMs of nascent proteins during membrane insertion (6). These findings, along with the observation that YidC increases insertion efficiency and/or stability of some membrane proteins, led to the view that YidC may act as a membrane-protein chaperone (24, 25).

In the present work we investigate the dynamics of lateral gate opening and closing in the SecYEG core translocon and in the SecYEG-YidC complex and address its role in cotranslational TM insertion. Fluorescence-based assays have revealed an important role of TM insertion in lateral gate opening (26), and structural details of the process have been studied using X-ray crystallography, cryo-electron microscopy, and computer simulations (27). At least three different lateral gate conformations have been structurally characterized—closed, partially open, and open—but it is unclear what role these conformations play during membrane protein insertion. Single-molecule fluorescence resonance energy transfer (FRET; smFRET) has proven insightful for studying the lateral gate of SecYEG, and previous work showed that the lateral gate samples at least three conformations during ATP-driven protein translocation by SecA (28). Here we employ a single-molecule fluorescence approach to study the dynamics of SecYEG in real time to understand how opening and closing of the lateral gate is coordinated during TM insertion.

## Results

### smFRET Reveals a Highly Dynamic Lateral Gate of SecYEG.

**Fig. 1.**
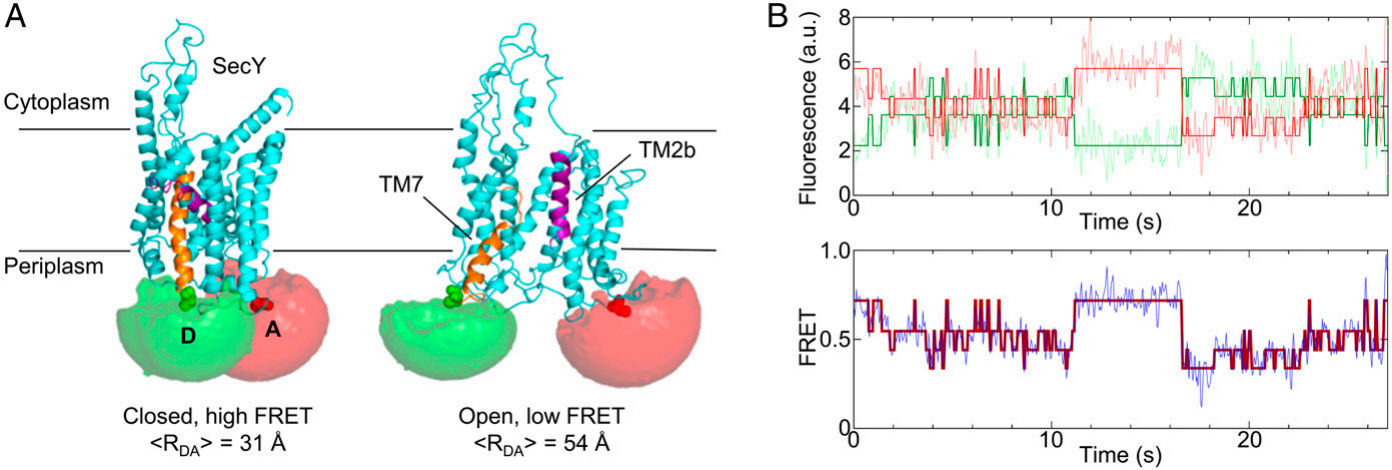
Single-molecule FRET labels to monitor lateral gate dynamics by TIRF. (*A*) Models of SecYEG in the closed (3J45.pdb) and open (3J00.pdb) conformations were used for coarse-grained simulations of donor (Cy3, green) and acceptor (Atto647N, red) fluorophores attached to cysteine side chains at positions 298 and 148 of SecY. The modeled accessible volumes predict an average distance between donor (D) and acceptor (A) of 31 Å (closed) or 54 Å (open). (*B*, *Upper*) Representative fluorescence trace from a single particle upon donor excitation. Donor fluorescence is plotted in light green and acceptor fluorescence in light red. The dark lines are idealized fits of the donor and acceptor traces. a.u., arbitrary units. (*B*, *Lower*) FRET trace computed from the donor and acceptor fluorescence in *Upper*. The red line represents the idealized FRET trace obtained from HMM.

In order to monitor the dynamics of the lateral gate of SecYEG in real time we have developed an smFRET approach using fluorescence-labeled translocon embedded in nanodiscs containing phospholipids of the bacterial membrane. A kinetically selective labeling strategy was employed to place a Cy3 donor fluorophore at position 298 in SecY and an Atto647N acceptor at position 148 (*SI Appendix*, Fig. S1*A*), chosen because the distance between these positions changes considerably between “open” and “closed” conformations of the lateral gate (8, 29). Nanodisc-embedded translocons labeled with Cy3 and Atto647N were functionally active as they were able to protect the nascent peptide of an RNC ligand from protease digestion to the same extent as wild-type, unlabeled SecYEG; fluorescence of donor or acceptor alone did not change upon ligand binding (*SI Appendix*, Fig. S1 *B* and *C*). To conceptualize the expected FRET changes, donor (Cy3) and acceptor (Atto647N) fluorophores were incorporated into structural models of SecYEG–nanodiscs with the lateral gate in either open or closed conformation. Possible dye positions were then sampled by accessible volume simulations (30), revealing an average donor–acceptor distance of 31 Å in the closed conformation and 54 Å in the open conformation (Fig. 1*A*). These distances are expected to lead to high and medium FRET, respectively, given a Förster radius of R_0_ = 51 Å for this label pair (31), and should exhibit a FRET decrease when the lateral gate transits from the closed to the open conformation.

**Fig. 2.**
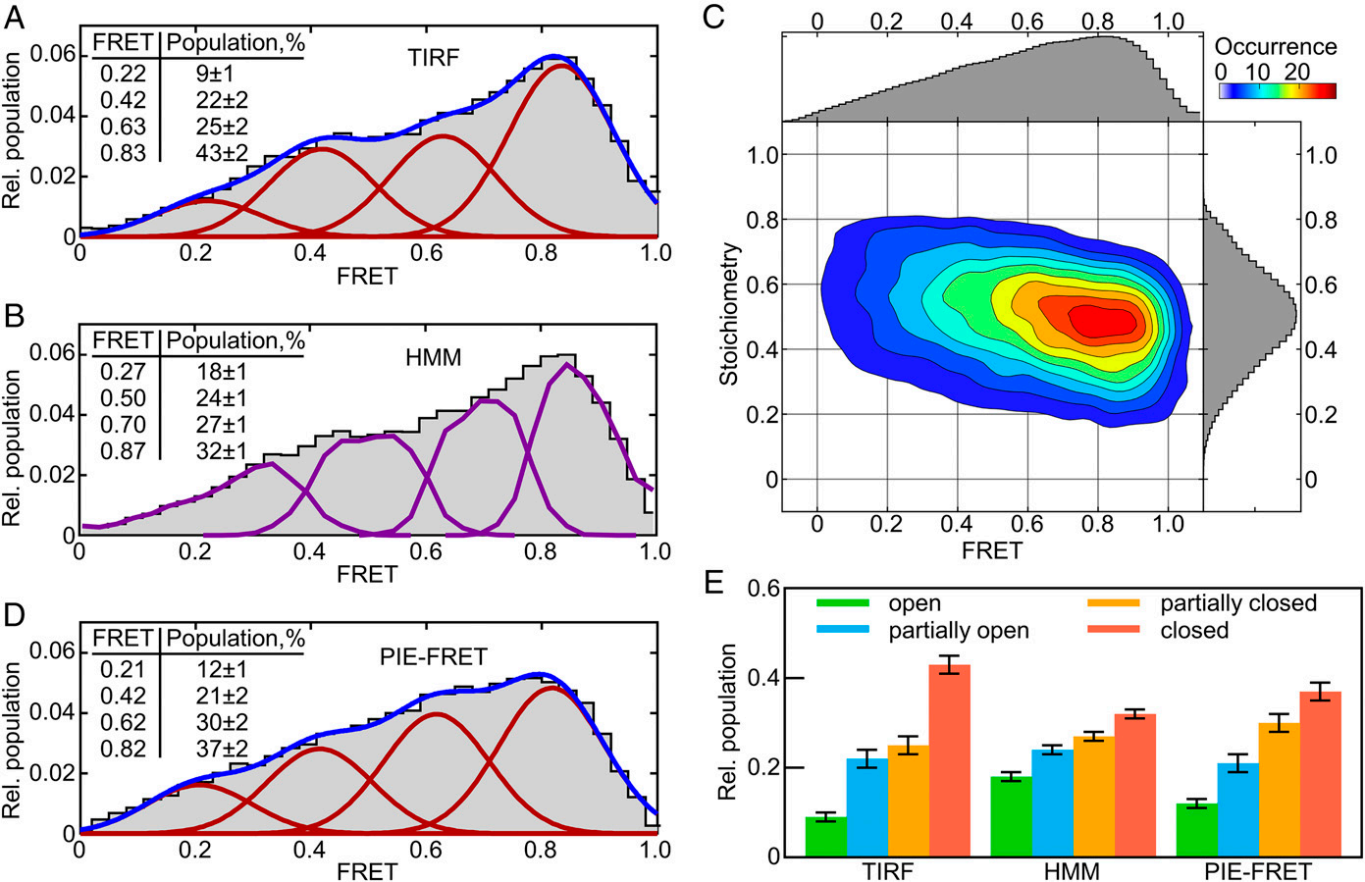
smFRET histograms of SecYEG fitted with four-state models. (*A*) FRET histogram obtained from immobilized nanodisc-reconstituted SecYEG complexes measured in TIRF. The blue curve indicates the cumulative distribution comprised of four Gaussian functions (red) obtained from fitting with σ = 0.1. (*Inset*) Mean FRET values and populations of FRET states along with SDs. (*B*) Same distribution as *A* fitted with a four-state HMM (purple). (*C*) Two-dimensional histogram of FRET efficiency vs. stoichiometry. Stoichiometry (S) is defined as S = n_D_/(n_D_ + n_A_) for each single molecule, where n_D_ is the number of donor fluorophores and n_A_ is the number of acceptor fluorophores. (*D*) FRET histogram obtained from freely diffusing nanodisc-reconstituted SecYEG complexes measured by PIE-FRET. The blue curve indicates the cumulative distribution composed of four Gaussian functions (red) obtained from fitting. (*Inset*) Mean FRET values and populations of FRET states. (*E*) Relative populations of the four FRET states calculated from the histograms in *A*, *B*, and *D*. FRET states from low to high FRET are labeled open (green), partially open (blue), partially closed (orange), and closed (red).

The dynamics of fluorescence-labeled SecYEG–nanodiscs were analyzed in a total internal reflection fluorescence (TIRF) microscope under donor excitation, with donor and acceptor fluorescence recorded on a charge-coupled device camera. The anticorrelated donor and acceptor time traces were used to calculate time-dependent FRET for individual molecules (Fig. 1*B*). Analysis of several hundred molecules reveals a broad FRET distribution (Fig. 2*A*), indicating a broad range of potential SecYEG conformations. Within this conformational landscape high-FRET states are favored, suggesting a trend toward a more closed lateral gate in the SecYEG–nanodisc complex in the absence of ligands.

Due to the broadness of the FRET histograms, there is no clear separation between discrete underlying FRET states. To quantify the conformational landscape we first sought to interpret the data in terms of several predominant states with their characteristic FRET values. We estimated the minimum number of states by testing models comprising two, three, four, or five Gaussian components. The statistical analysis suggests that the four-state model is optimal (*SI Appendix*, Fig. S2 and Table S1), with average FRET values ranging from 0.22 to 0.83, each with an SD σ = 0.1. This analysis suggests that the conformational landscape of the lateral gate is not simply open vs. closed but requires a model with at least four discrete conformational states. The high-FRET state is predominant (43%), again indicating that the vacant lateral gate tends to be in the closed state. This result is consistent with previous smFRET experiments performed with FRET labels on the cytoplasmic side of the lateral gate, which were interpreted using a Gaussian model containing three broad FRET states (σ = 0.16 to 0.31) (28). The previous study found a very strong preference for the closed conformation in the absence of ligands (80% population). Difference in the number of populations identified in Gaussian fitting (three in the previous study vs. four here) is likely related to the very different σ values, since σ values up to 0.31 in the previous study (compared to σ = 0.1 here) could indicate multiple, unresolved FRET states.

**Fig. 3.**
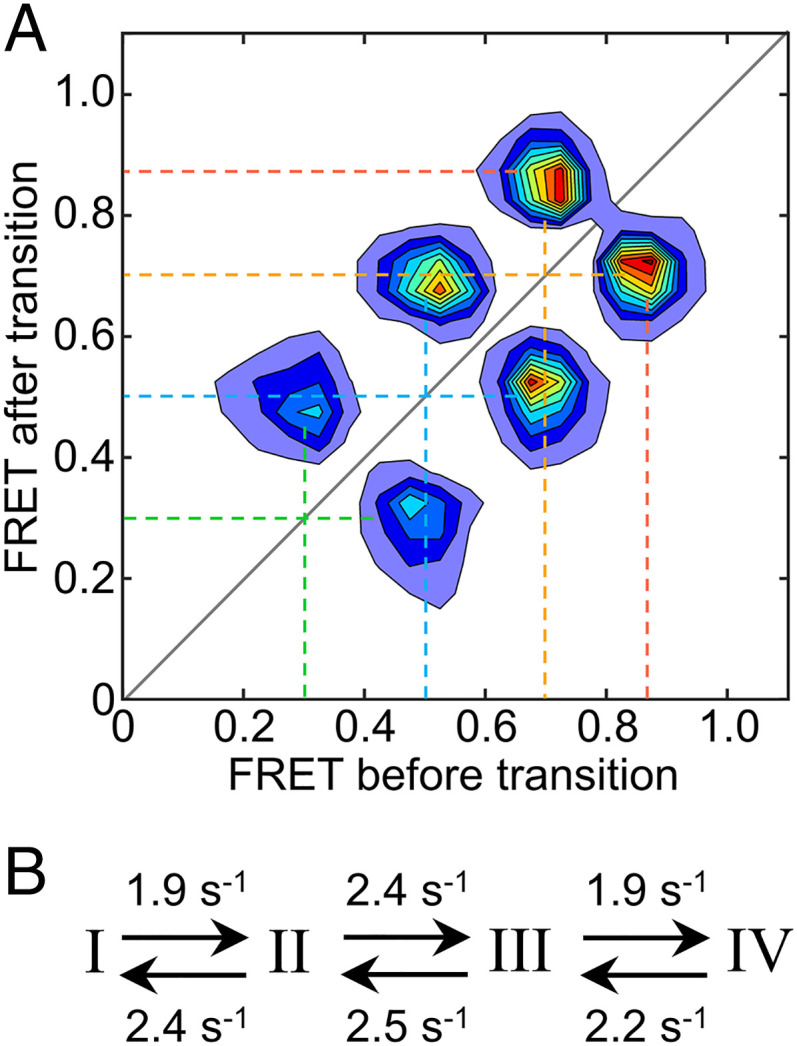
Kinetic analysis of lateral gate opening by HMM. (*A*) Transition density plot indicating the average FRET before and after each transition identified by four-state HMM analysis. (*B*) Associated kinetic mechanism.

Next, we analyzed the smTIRF data with hidden Markov modeling (HMM) using vbFRET software (32). The fitted four-state HMM indicates FRET states up to 0.87, the latter with a population around 32% (Fig. 2*B*). Both Gaussian and HMM fitting, therefore, predict that the highest FRET state is preferred, albeit with a somewhat different distribution between the states. Kinetic analysis of the four-state HMM reveals a linear kinetic mechanism connecting the four FRET states from lowest to highest, and all rate constants are similar, in the range 1.9 to 2.5 s^−1^ (Fig. 3 and *SI Appendix*, Table S2). We have also explored larger numbers of states using HMM fitting and found that, regardless of how many are modeled, FRET states are connected by a linear kinetic mechanism with rate constants in the range 1 to 10 s^−1^ (*SI Appendix*, Fig. S3 and Table S2). To probe the possibility of additional fast-timescale dynamics at the lateral gate of SecYEG, we carried out pulsed interleaved excitation (PIE) FRET experiments with a 100-ns time resolution by measuring freely diffusing translocons in a confocal fluorescence microscope. Since this method is particularly sensitive to bleed-through of donor emission into the acceptor detection channel, for these experiments we prepared double-labeled SecYEG in nanodiscs with Atto488 as FRET donor (position 148) and Atto647N as acceptor (position 298). This donor–acceptor pair provides better spectral separation than Cy3/Atto647N and limits bleed-through to 1.8%, compared to 13% for Cy3/Atto647N. PIE-FRET experiments allow us to measure the stoichiometry of donor and acceptor fluorophores for each particle and select only those with one donor and one acceptor for further analysis (*Materials and Methods*).

**Fig. 4.**
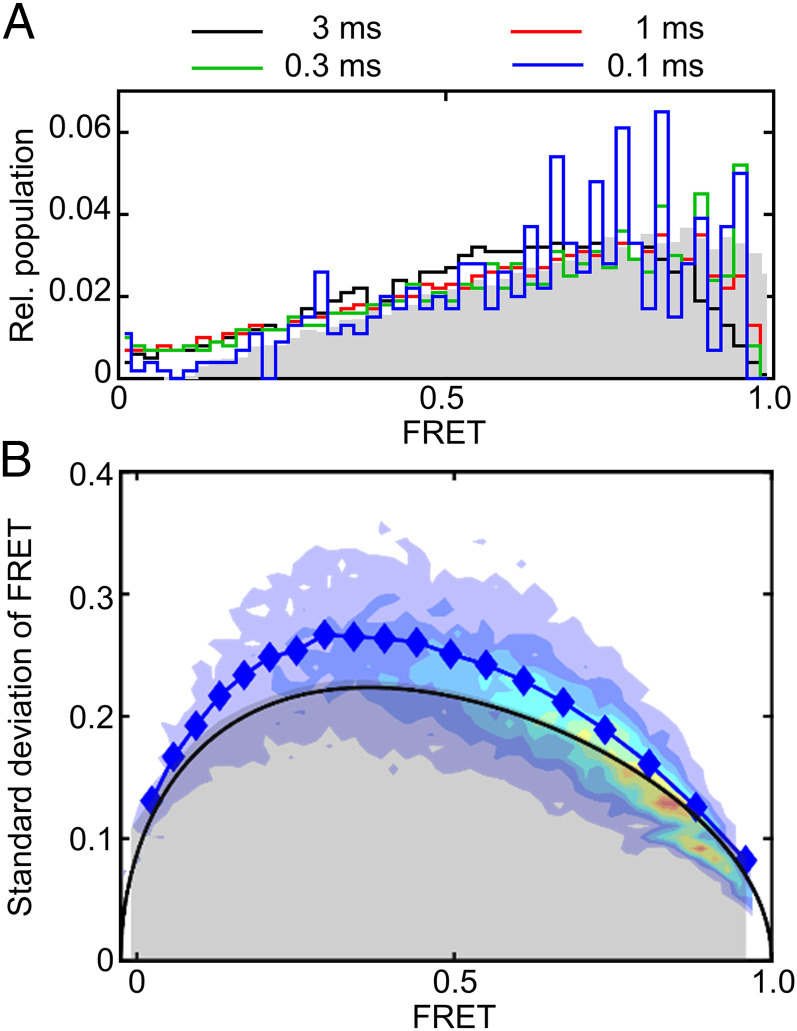
PIE-FRET analysis of freely diffusing nanodisc-reconstituted SecYEG. (*A*) FRET histograms were computed after dividing each burst into time windows of fixed length (colored lines). The raw FRET histogram is plotted for comparison (gray). (*B*) BVA of the PIE-FRET data were performed by computing the SD of FRET, σ(FRET), for each burst (contour) and averaged over specific FRET intervals (blue symbols). The gray shaded area represents the 99.9% confidence interval computed assuming static FRET states.

The FRET histogram obtained from PIE-FRET experiments closely resembles that obtained from TIRF, despite the use of a different donor fluorophore, and improved time resolution (Fig. 2*D*). In generating this histogram the average FRET over the entire diffusion time (τ_D_ = 0.7 ms on average) is computed for each molecule. Examination of fast-timescale dynamics, however, requires investigating the possibility that each molecule visits different FRET states while diffusing through the focal volume. To this end, we performed a time-window analysis, in which the FRET data from each molecule were partitioned into fixed-length time windows in the range 0.1 to 3 ms (Fig. 4*A*). If transitions between different FRET states occur on the same timescale or slower than these time windows, then this analysis will effectively sort data into individual FRET states and cause the FRET histogram to resolve into multiple, narrow FRET peaks. For SecYEG, however, the broad FRET histogram persisted at all time windows and did not resolve into more narrow states, indicating that no FRET transitions are evident on timescales of 0.1 to 3 ms (Fig. 4*A*).

To investigate the possibility of faster dynamics, we carried out a burst-variance analysis (BVA) on the PIE-FRET data by computing the SD of FRET values obtained for each molecule while inside the confocal volume (*Materials and Methods*). If a single molecule occupies more than one FRET state while diffusing through the confocal volume, then some fluorescence emissions will result from lower FRET and some from higher FRET, such that the SD in FRET will be larger than expected for a single FRET state. We find that SDs in FRET efficiencies are significantly higher for SecYEG (blue symbols, Fig. 4*B*) than what is predicted assuming static FRET states (black line and gray shaded area, Fig. 4*B*). Thus, BVA suggests dynamic FRET changes at the lateral gate of SecYEG during the diffusion time (τ_D_ = 0.7 ms; *SI Appendix*, Fig. S4*C*) of the experiment. Although this analysis cannot identify the precise timescale or origin of the dynamics, photophysical effects including triplet state, multiple acceptor states, and photobleaching were ruled out as possibilities (*SI Appendix*, Fig. S4 *B*–*D*). Thus, the large SD in FRET identified by BVA likely results from structural dynamics at the lateral gate of SecYEG.

Fast-timescale FRET measurements (PIE-FRET) suggest that submillisecond timescale dynamics at the lateral gate of SecYEG contribute to the broadness of the FRET histogram. Since the fluorescent probes are located in loop regions adjacent to the lateral gate helices, it is not clear how the loops vs. the lateral gate helices themselves contribute to the submillisecond dynamics. HMM analysis of the TIRF data, however, provides some insight by identifying linear kinetic mechanisms for both four-state and higher-order models (Fig. 3 and *SI Appendix*, Fig. S3 *D* and *E*). If the dynamics of loops and helices at the lateral gate were completely uncoupled, then a nonlinear kinetic mechanism would be expected, with different HMM states corresponding to different combinations of loop/helix conformations. Instead, the kinetic mechanism is linear, indicating that if HMM states do indeed correspond to different combinations of loop/helix conformations then conformational changes in loops and helices at the lateral gate are coupled. Consistent with this finding, examination of all seven available translocon structures from *Escherichia coli* in which the loops were resolved has revealed that the distance between lateral gate TMs is indeed correlated with the distance between the FRET label positions, which are in loop regions (*SI Appendix*, Fig. S5). The resolution of the available structures (5 to 14 Å), however, does not permit a more detailed comparison of loop movements in these structures.

### Quantifying Changes at the Lateral Gate.

**Fig. 5.**
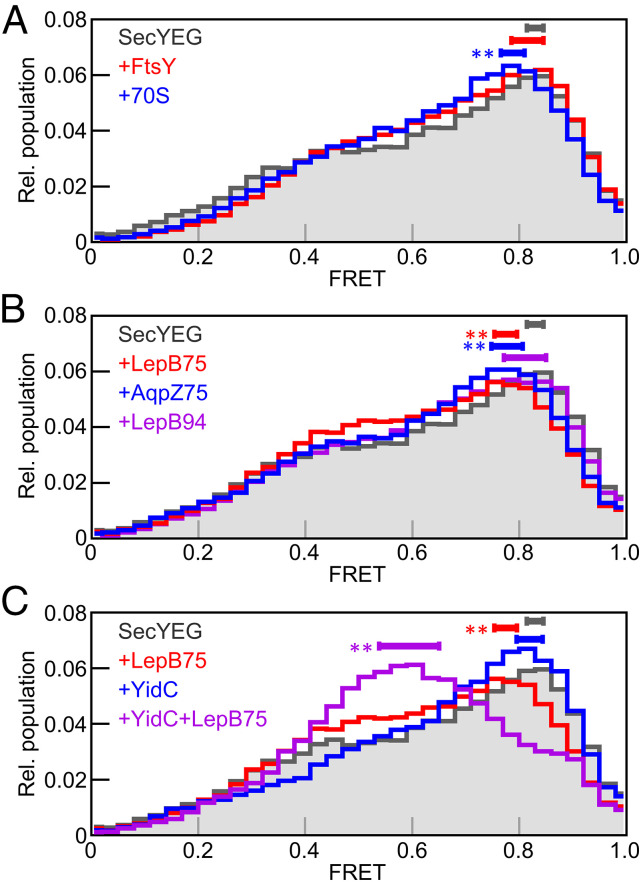
Effect of ligand binding on smFRET of SecYEG. (*A*) Overlay of FRET histograms obtained for SecYEG alone (gray area), with 70S ribosomes (blue), or with SRP receptor, FtsY (red). The peak range for each dataset, calculated by Monte Carlo simulation, is indicated by a solid bar above the histogram. Significant differences compared to SecYEG alone are indicated (**P < 0.001). (*B*) Same as *A* but with LepB75-RNC (red), LepB94-RNC (blue), or AqpZ75-RNC (purple). (*C*) Same as *A* but with YidC (blue), LepB75-RNC (red), or YidC+LepB75-RNC (purple).

In order to understand how the conformation of the lateral gate changes during membrane protein insertion, we performed smFRET experiments in the presence of different ligands that bind to the translocon. Based on the previously measured affinities of these ligands for binding to SecYEG (26, 33), we have used ligands at sufficiently high concentrations to ensure saturation (*Materials and Methods*). We first tested the effect of the SRP receptor, FtsY, since it was previously shown that FtsY binds to SecYEG at the lateral gate (34), where it is activated for recruitment of SRP in complex with translating ribosomes (33). We find that binding of FtsY to SecYEG causes a decrease in the low-FRET region of the histogram and an increase in the medium-FRET region, although the changes are small (Fig. 5*A*). To quantify these changes we compared Gaussian and HMM fits of the FRET histograms obtained with and without FtsY and reached two different, nonexclusive interpretations. The Gaussian model indicates a 6% decrease in the low-FRET state and a 6% increase in the medium-high-FRET state (both of which are statistically significant). By contrast, HMM fitting does not identify any significant changes in state populations, but rather a shift from low and medium-low FRET states toward higher FRET (*SI Appendix*, Figs. S6 and S7).

In order to avoid misinterpretation of the data by arbitrarily selecting a Gaussian vs. HMM model, we have developed a model-free approach to compare smFRET datasets based on statistically robust, nonparametric analyses. By directly comparing the FRET histograms obtained in the absence and presence of FtsY using a Kolmogorov–Smirnov test, we identify a significant difference in the FRET histograms (*P* < 0.001). The effect of FtsY binding is a slight tendency to increase FRET due to a shift of low FRET values toward medium FRET. However, the FRET peak does not change significantly when FtsY is bound to SecYEG (horizontal bars in Fig. 5*A*), indicating that the ability of FtsY to shift the lateral gate to more closed is very small. When 70S ribosomes bind to the translocon we observe a significant change in the FRET histogram (*P* = 0.0025) and a significant shift in the FRET peak toward lower FRET, indicating a trend toward lateral gate opening. Analysis of the FRET histograms using Gaussian, HMM, or model-free approaches provides the same principal interpretation of the data with regard to lateral gate opening (Fig. 5 and *SI Appendix*, Figs. S6 and S7). In the following, we focus on the model-free analysis of FRET histograms, because it provides straightforward statistical comparisons and is independent of the number of FRET states used for analysis. For completeness, the results of Gaussian and HMM fitting are reported for all data in *SI Appendix*, Figs. S6 and S7, respectively.

### Lateral Gate Opening upon TM Insertion into SecYEG.

To investigate changes at the lateral gate during TM insertion we added RNCs bearing the first 75 amino acids of the inner-membrane protein LepB (LepB75-RNC). The nascent chain in this complex is long enough for the N-terminal hydrophobic TM1 of LepB to be exposed and inserted into the translocon in an N-out orientation (26, 35); the complete occupancy of the translocon was validated by biochemical experiments (*SI Appendix*, Fig. S8). Binding of these RNCs to the translocon causes a significant change in the histogram (*P* < 0.001) and shifts the peak toward lower FRET (Fig. 5*B*). When the nascent chain of LepB is extended by 19 amino acids (LepB94-RNC), the FRET histogram shifts back toward higher FRET, suggesting lateral gate closing with longer nascent chain (Fig. 5*B*), while biochemical controls confirm that both RNCs interact with translocon to the same extent (*SI Appendix*, Fig. S8). When a different nascent membrane protein, AqpZ75-RNC, is bound to the translocon a significant change in the FRET histogram is observed compared to no ligand (*P* = 0.0025), and a decrease in the peak FRET value indicates lateral gate opening.

### Effect of YidC on Lateral Gate Dynamics.

Next, we have studied the effect of YidC on the lateral gate of SecYEG. YidC, itself a transmembrane protein, interacts with SecY at the lateral gate (23) and is a component of the holotranslocon (36). YidC induces a change in the FRET histogram (*P* < 0.001), driven by a decrease in the shoulder of the FRET histogram at 0.4 (Fig. 5*C*). While this indicates a tendency to close the lateral gate, the peak FRET value remains unchanged. When LepB75-RNC is added to the SecYEG–YidC complex there is a further change in the histogram (*P* < 0.001), with a large shift of the peak FRET value from 0.8 to 0.6, consistent with lateral gate opening (Fig. 5*C*). The effect of TM insertion is clearly stronger than that observed in the absence of YidC, which suggests that the interaction of YidC with the TM of a nascent protein induces an open state of the lateral gate.

### Amino Acid Exchanges in the Lateral Gate Alter Conformational Dynamics of SecYEG.

**Fig. 6.**
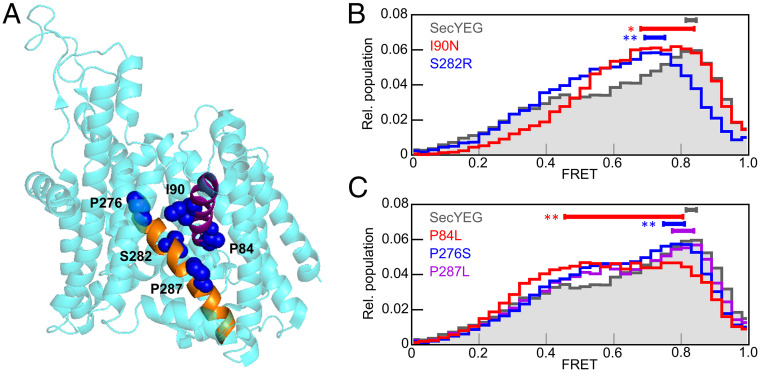
smFRET histograms from SecYEG gate variants. (*A*) Positions of lateral gate mutations. Model of SecYEG (cyan) is shown as a cartoon, and lateral gate amino acids that were mutated in this study are numbered shown as blue spheres. Lateral gate helices TM2 and TM7 are colored purple and orange, respectively. (*B*) Overlay of FRET histograms obtained with wild-type SecYEG (gray area) and prl suppressor variants. The peak range for each dataset is indicated by a solid bar above the histogram. Significant differences compared to SecYEG alone are indicated (**P < 0.001; *P < 0.05). (*C*) As in *B*, but for cold-sensitive variants, P84L and P276S, as well as the export-deficient variant P287L.

A number of single amino acid exchanges in SecY were previously shown to cause phenotypic effects in bacteria, including cold sensitivity, compromised membrane insertion, and disturbed protein secretion (13). We have performed smFRET analyses with five different single amino acid substitutions, P84L and I90N in TM2 as well as P276S, S282R, and P287L in TM7, which all map to the lateral gate of SecYEG (Fig. 6*A*) and cause phenotypic effects in vivo. The I90N and S282R variants were identified as *prlA* suppressors, which support secretion of reporter proteins with mutant signal sequences (37, 38). Amino acid exchange P287L, on the other hand, caused a deficiency in secretion of a reporter construct (39), while P84L and P276S substitutions were identified based on their cold-sensitive phenotypes (40, 41). The *prlA* suppressors, I90N and S282R, both cause changes in the FRET histogram (*P* < 0.001) and cause the FRET peak to shift to lower values, indicative of lateral gate opening (Fig. 6*B*). The two cold-sensitive mutants, P84L and P276S, also exhibit significant changes in the FRET histograms with shifts of the peak toward lower FRET (Fig. 6*C*). The P287L substitution, on the other hand, had no effect on the FRET histogram, indicating that not all substitutions at the lateral gate influence the propensity to open. Interestingly, both mutations in TM2 (P84L and I90N) result in very broad FRET peaks due to the flatness of the histograms near the peak.

## Discussion

The present smFRET experiments with nanodisc-embedded double-labeled translocons reveal that the translocon is rather dynamic and fluctuates over the whole range between open and closed states. Analysis of population distribution and kinetics of transitions suggests that the conformational landscape at the lateral gate of SecYEG is better described by a continuum, rather than by defined conformations. The FRET distributions measured at the single-molecule level correspond to functionally relevant conformations of the lateral gate as evidenced by the changes observed upon RNC binding, which induces the anticipated lateral gate opening (10, 26, 42). These measurements provide insight into how lateral gate dynamics change at different stages during the insertion process and reveal a strong effect of YidC in stabilizing the open conformation of the lateral gate upon insertion of a hydrophobic TM. Large changes in lateral-gate fluctuations are also observed upon introduction of mutations that carry well-characterized phenotypes in vivo, providing evidence for the importance of lateral-gate dynamics for cellular homeostasis.

A number of studies have reported structural details of the translocon in different functional states. We have carried out a comprehensive survey of lateral gate opening in the models available from these reports and note, first, that the distance between lateral gate helices TM2 and TM7 varies widely in the different structures (*SI Appendix*, Fig. S5 *B* and *C*). While the technical details underlying these reconstructions—bacterial vs. archaeal vs. eukaryotic translocons, detergent- vs. membrane-stabilized TMs, and ligand-free vs. ribosome-bound vs. RNC-bound—also vary significantly between the different studies, the ability of the lateral gate to open to different extents has been clearly established (8, 10, 42–45). We have found that even after grouping these models according to which ligand is bound (no ligand, ribosome-bound, or RNC-bound) a wide range of lateral gate conformations is observed within each group (*SI Appendix*, Fig. S5 *B* and *C*). Notably, lateral gate opening is not exclusively observed in RNC-bound structures; partial opening of the lateral gate has been observed in the absence of ligands (albeit induced by crystal packing with a neighboring copy of SecY) (46), in a SecYEG–SecA complex (47), and in a Sec61–ribosome complex (43). Taken together, these models tend to suggest a relatively flat conformational landscape of lateral gate fluctuations. On the whole, the large variance in conformations observed in translocon structures supports our present finding that the lateral gate is highly dynamic, both in the presence and absence of ligands.

TIRF experiments indicate that transitions between different lateral gate conformations occur multiple times every second. For comparison, the ribosome requires about 1 or 2 s to produce a 20-amino-acid TM, given a translation rate of 10 to 20 amino acids in *E. coli*. Thus, intrinsic dynamics of the lateral gate are fast enough to permit stochastic insertion of a TM into the phospholipid bilayer during synthesis, without the need for large changes in lateral gate kinetics. The existence of additional, submillisecond dynamics at the lateral gate revealed by PIE-FRET likely reflects interconversion between subconformations of the lateral gate and results in broadening of the FRET states observed upon averaging over the diffusion time (PIE-FRET) or exposure time (TIRF), depending on the smFRET method.

**Fig. 7.**
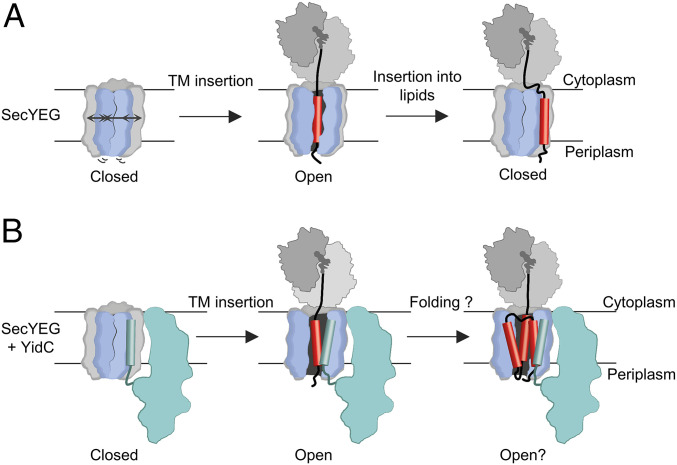
Models of lateral gate opening and closing during TM insertion. (*A*) Model of TM insertion via SecYEG. The lateral gate is mostly closed before TM insertion, more open during TM insertion, and closes again after extension of the nascent chain. (*B*) Model of TM insertion facilitated by SecYEG and YidC. The lateral gate is mostly closed before TM insertion. During TM insertion, YidC interacts with the TM and holds the lateral gate open, which may facilitate insertion/folding of polytopic membrane proteins.

In the resting state, when no ligand is bound to SecYEG, the lateral gate is predominantly closed, with the majority of translocon molecules displaying high FRET, consistent with previous data (28). The FRET changes we observe upon addition of different ligands reveal how lateral gate dynamics change during insertion of nascent proteins into the membrane (Fig. 7). In order to compare these different complexes, we have developed a model-free approach to smFRET analysis, which avoids interpretation of the data using a model with an arbitrary number of states. When vacant 70S ribosomes are bound to the translocon the lateral gate shifts slightly toward more open states, consistent with previous ensemble fluorescence measurements (26). This is also consistent with the structure of a eukaryotic ribosome-bound translocon complex which indicates partial opening of the lateral gate (43). When RNCs bind to the translocon, insertion of the hydrophobic TM1 of LepB shifts the conformational landscape of the lateral gate toward open. We have previously shown that during cotranslational insertion of LepB, TM1 is inserted with a stable N-out orientation at nascent chain lengths of 75 and 94 amino acids (35). In the present study we find that, at a length of 75 amino acids, the TM1 of LepB favors lateral gate opening. Similar changes at the lateral gate were observed when another membrane protein, AqpZ75-RNC, was tested. When the nascent chain of LepB is extended from 75 to 94 amino acids, however, the conformational landscape of the translocon shifts again in the direction of closed and is not significantly different from that observed in the absence of RNC. This indicates that TM1 of LepB94 is less often at the lateral gate and would be consistent with lateral movement into the lipid bilayer (Fig. 7). Closing the lateral gate upon lipid insertion of TM1 in an N-out orientation may help direct the nascent chain connecting TM1 and TM2 to the correct, cytosolic, side of the membrane during ongoing translation. Here, TM1 may interact with the “outside” of the lateral gate, which has been suggested for other substrates (9, 12). The lateral gate, however, remains dynamic during and after insertion of TM1.

Changes in the conformational landscape of the lateral gate are far more pronounced in the presence of YidC, which contacts the lateral gate directly via its N terminus (21). Consistent with that report, we find that YidC tends to enrich the high-FRET region of the histogram, indicating that it restricts lateral gate dynamics to some extent. Upon binding of LepB75-RNC, however, there is a dramatic shift in the conformational landscape toward an open lateral gate. This opening is much larger than what is observed in the absence of YidC and may result from an association of LepB TM1 with YidC in the lateral gate (Fig. 7), consistent with the finding that YidC interacts with TMs of nascent membrane proteins during membrane insertion (6, 21, 48). Additionally, recent work has indicated that folding of the polytopic membrane protein LacY in a phospholipid bilayer is altered by interaction with YidC (49), in line with the proposed role of YidC as a membrane protein chaperone (50, 51). With this in mind, it seems likely that by interacting with TMs of nascent proteins in the lateral gate YidC can assist in the insertion and folding of polytopic membrane proteins. We note that of the four membrane proteins known to rely on SecYEG and YidC for insertion in *E. coli*, FoA (20), NuoK (52), and CyoA (53) contain multiple TMs, while FoB (54) contains a single, long TM. All of these membrane proteins, however, are components of multisubunit membrane protein complexes, suggesting that YidC may also play a role in aiding the assembly of larger complexes within the membrane.

We have also tested how the lateral gate helices themselves (TM2 and TM7) constrain the conformational landscape of the lateral gate by testing SecYEG variants. In general, we find that mutations in the gate helices can have large effects on the conformational landscape of the lateral gate, but no mutation tested caused the lateral gate to remain exclusively open or closed in the absence of ligands. The viability of each mutant tested suggests that a relatively flat conformational landscape is an important property of the lateral gate and is required for proper function. Amino acid substitutions I90N and S282R both confer a *prl* suppressor phenotype by allowing secretion of proteins with mutated signal-anchor sequence, and we find that both of these substitutions shift the conformational landscape of the lateral gate toward more open. Lateral gate opening was shown to promote protein secretion, since covalent cross-linking of the lateral gate with a short-arm cross-linker inhibited SecA-mediated protein translocation (19). A more open lateral gate, induced by I90N or S282R mutation, would likely be prone to promiscuous insertion of mutant signal-anchor sequences, consistent with the phenotype.

The cold-sensitive substitutions also shift the conformational landscape toward more open and, while it is not possible to predict how this culminates in a cold-sensitive phenotype, we do find a common structural basis for this effect. In models of SecYEG in the closed conformation, Pro84 and Pro276 point away from the lateral gate toward neighboring helices TM3 and TM8, respectively (Fig. 6*A*). This suggests that the P84L substitution disrupts the packing of TM2 with TM3 and destabilizes the closed conformation. The P276S substitution is less severe, probably owing to the substitution with a small, unbranched amino acid. Destabilization of the closed conformation might, therefore, be important to permit opening of the lateral gate and TM insertion at low temperatures, which favor a closed lateral gate (26). Interestingly, we find that both substitutions in TM2 (I90N and P84L) induce dramatic flattening of the FRET histogram and result in very broad FRET peaks. This finding suggests that in these variants the potential energy surface for the gate fluctuations is flat, with closed conformations not significantly favored over partially open conformations. The lateral gate helix TM2 may, therefore, play an important role in stabilizing the closed lateral gate and prevent exposure of the hydrophobic lipids to the surrounding aqueous environment.

In summary, our results demonstrate remarkable conformational flexibility in the lateral gate of SecYEG throughout the insertion of membrane proteins. Even in the absence of ligands the lateral gate samples multiple conformations that are open or closed to different extents. This is likely an important feature of the translocon, allowing it to open or close at different stages of membrane insertion, and for TMs of varying sequence. We observe fluctuations at the lateral gate of SecYEG at both fast (submillisecond) and slow (subsecond) timescales, with the slow conformational changes still rapid enough to keep up with the rate of cotranslational membrane protein insertion. Insertion of a TM shifts the conformational landscape of the lateral gate toward open, which is expected because it facilitates TM integration into the phospholipid bilayer. When YidC is present there is a strong tendency toward lateral gate opening during TM insertion, which may aid in the insertion and folding of nascent polytopic membrane proteins. The lateral gate continually samples a wide spectrum of conformations throughout the process of TM insertion, indicating that transmembrane proteins insert and fold in a dynamic environment.

## Materials and Methods

### Materials.

Fluorescence measurements were performed in buffer A (50 mM Tris, pH 7.5, 70 mM NH_4_Cl, 30 mM KCl, and 7 mM MgCl_2_) at 22 °C. Proteins were recombinantly expressed and purified following established protocols (26, 33). RNCs were prepared by in vitro translation and purified by centrifugation through a sucrose cushion (26). Translation efficiencies were better than 70%. Double labeling of SecYEG(148/298) was achieved by kinetically selective labeling of position 298 with Cy3-maleimide (Lumiprobe) and 148 with Atto647N-maleimide (Atto-Tec). The final material contained 0.5 molecules of Cy3 (donor) and 1.3 molecules of Atto647N (acceptor) per SecYEG. Atto488/Atto647N–labeled SecYEG(148/298) was prepared similarly. Accessible volume simulations of Cy3 (donor) and Atto647N (acceptor) fluorophores were performed using the FRET Positional Screening (FPS) software (30).

Nanodiscs containing functional SecYEG (26, 35, 55) were prepared from purified SecYEG, biotin-coupled MSP1D1 protein, and total *E. coli* phospholipids (Avanti Polar Lipids) according to published protocols (26). Nanodiscs containing SecYEG and YidC were prepared as above with addition of YidC at a concentration equal to that of SecYEG. The ratio of YidC:SecY in nanodiscs was ∼1:1 (*SI Appendix*, Fig. S9*A*). Assembly of YidC to SecY in the same nanodisc was confirmed by cross-linking and Western blotting using an anti-YidC antibody (a gift from H. G. Koch, University of Freiburg, Germany) (*SI Appendix*, Fig. S9*B*). The activity of nanodisc-embedded SecYEG labeled with Cy3 and Atto647N was confirmed by testing the ability to protect radiolabeled nascent chain in an RNC from digestion by proteinase K (PK) (*SI Appendix*, Fig. S1).

### smFRET Experiments Using TIRF.

All smFRET experiments were performed at 22 °C in buffer A with additions. For experiments with translocon ligands, RNCs [100 nM, dissociation constant (*K*_d_) = 10 nM (26)], 70S ribosomes [100 nM, *K*_d_ = 20 nM (26)], or FtsY [2 µM, *K*_d_ = 0.2 µM (33)] and 5′-guanylyl imidodiphosphate (GDPNP; 0.5 mM) were added to the imaging buffer. Nanodiscs containing biotin-linked MSP1D1 protein were immobilized on biotin-polyethylene glycol–functionalized coverslips according to published protocols (56). TIRF imaging was performed on an IX 81 inverted microscope (Olympus) using 561-nm solid-state laser excitation (25 mW) and fluorescence time courses for donor (Cy3) and acceptor (Atto647N) were extracted as previously described (56). Anticorrelated fluorescence traces (correlation coefficient <−0.4) that contained single photobleaching steps for acceptor and then donor were selected for further analysis. The acceptor fluorescence was corrected for bleed-through of donor signal into the acceptor channel. Each trajectory was then smoothed once over three data points. FRET efficiency was corrected for relative quantum yields and detection efficiencies of donor and acceptor. FRET-histograms were fitted to Gaussian distributions using GraphPrism. The vbFRET software package (http://vbfret.sourceforge.net/) (32) was used for HMM analysis of the FRET data and stochastic rate constants were determined by dwell-time analysis of the idealized FRET traces (57).

All model-free analysis was performed in MATLAB. FRET histograms obtained from TIRF experiments were compared using the Kolmogorov–Smirnov test to quantify the largest difference in the empirical cumulative distribution functions obtained from two experiments (58). For significance testing, 1,000 simulated datasets were constructed for each experiment by empirical Monte Carlo simulation. Similarly, the range of the FRET peak for each experiment was determined by identifying the peak FRET value in each of the 1,000 simulated datasets.

### PIE-FRET Experiments.

PIE-FRET was performed using the MicroTime 200 system (PicoQuant). Alternating 485-nm and 640-nm laser excitation (PIE mode) was carried out at 20 MHz with laser powers 80 and 12 µW, respectively. Fluorescence-labeled nanodiscs were measured at 22 °C with a concentration adjusted to yield an average of less than 0.1 molecules within the confocal detection volume. Each measurement was performed for 15 min using freshly diluted sample. A total of 21 measurements (5.25-h measurement time) were compiled for data analysis. PIE-FRET data were analyzed using PIE analysis with MATLAB (PAM) software (59). The population with one donor and one acceptor (stoichiometry = n_D_/(n_D_ + n_A_) = 0.5) was selected for further analysis (17,000 molecules) and corrected for relative quantum yields (Φ) and detection efficiencies (*g*) of donor and acceptor. Time-window analysis was performed by dividing each burst into time windows with lengths of 3, 1, 0.3, or 0.1 ms and computing the resulting FRET histogram for time windows with more than 25 photons (*D*_ex_*D*_em_ + *D*_ex_*A*_em_) (Fig. 4*A*). BVA was performed using PAM software (59) in order to investigate the possibility of dynamic changes at the lateral gate of SecYEG, while diffusing through the confocal volume (Fig. 4*B*).

## Supplementary Material

Supplementary File

## Data Availability

All study data are included in the article and/or *SI Appendix*.
