# Supplementary material for: Lateral gate dynamics of the bacterial translocon during cotranslational membrane protein insertion
Source: Proc Natl Acad Sci U S A. 2021 Jun 23;118(26):e2100474118. doi: 10.1073/pnas.2100474118 (PMC8256087; doi:10.1073/pnas.2100474118)
Supplement: Supplementary File [file pnas.2100474118.sapp.pdf]

# Supporting Information

# Lateral gate dynamics of the bacterial translocon during cotranslational membrane protein insertion

Evan Mercier[1†], Xiaolin Wang,[1†] Manisankar Maiti,[1] Wolfgang Wintermeyer[1], and Marina V. Rodnina[1*]

[1]Max Planck Institute for Biophysical Chemistry, Department of Physical Biochemistry, 37077 Göttingen, Germany

[†] These authors contributed equally to this work.

*Correspondence: rodnina@mpibpc.mpg.de

This file includes:

Supporting Materials and Methods

Supporting Figures S1 to S9

Supporting Tables S1 and S2

Supporting References

## Materials and Methods

**Materials.** Fluorescence measurements were performed in buffer A (50 mM Tris, pH 7.5, 70 mM $NH_4Cl$, 30 mM KCl, 7 mM $MgCl_2$) at 22°C. SecYEG variants with cysteine at SecY position 148, 298, or both (SecYEG(148), SecYEG(298), or SecYEG(148/298), respectively) were prepared by site-directed mutagenesis of Cys-less SecYEG (1, 2). The plasmid encoding the double cysteine variant was then used as template for site-directed mutagenesis to introduce single amino-acid substitutions at the lateral gate (P84L, I90N, P276S, P287L, and S282R). The single-cysteine variant MSP1D1(Cys2) was prepared by site-directed mutagenesis of wild-type MSP1D1. Genes encoding LepB and AqpZ were PCR-amplified from *E. coli* (DH5α) genomic DNA, cloned into pET24a vector by ligation-independent cloning. DNA constructs were confirmed by sequencing (SeqLab). SecYEG, FtsY and MSP1D1(Cys2) were recombinantly expressed and purified following established protocols (2, 3). YidC was expressed and purified following the protocol used for SecYEG with minor modifications. Fractions from a HisTrap FF column (GE Healthcare) containing YidC were dialyzed against buffer B (50 mM sodium acetate, pH 5.3, 5 mM $MgCl_2$, 10% (v/v) glycerol, 0.03% N-dodecyl-beta-maltoside (DDM; Thermo Fisher Scientific)) at 4°C overnight. Soluble protein post dialysis was concentrated and re-buffered into buffer C (20 mM HEPES, pH 7.5, 70 mM $NH_4Cl$, 30 mM KCl, 7 mM $MgCl_2$, 10% (v/v) glycerol) on a PD-10 column (GE Healthcare). Biotin coupling to MSP1D1(Cys2) was carried out in buffer C with 10-fold excess biotin-maleimide (Sigma-Aldrich) for 2 h at 22°C prior to quenching with 2-mercapto ethanol (2-ME) and removal of unreacted biotin-maleimide by gel filtration (HiLoad Superdex 75 26/60, GE Healthcare).

## Methods

**Preparation of donor/acceptor-labeled SecYEG.** Labeling reactions were carried out at 22°C in buffer D (20 mM HEPES, pH 7.0, 150 mM KCl, 10% (v/v) glycerol, 0.03% DDM). To selectively label one position in SecY with donor and another with acceptor, we took advantage of the finding that different positions in SecY are more or less prone to the incorporation of fluorescent maleimide derivatives (4). Reaction time courses with sulfo-cyanine3-maleimide (Cy3-maleimide; Lumiprobe) were carried out to compare single-cysteine variants of SecYEG using 75 µM SecYEG and 150 µM Cy3-maleimide. Labeling time courses were obtained by quenching aliquots of the labeling reaction in SDS-loading buffer containing 1% 2-mercapto ethanol (2-ME) and separating labeled proteins by SDS-PAGE prior to imaging (FLA-7000 Fujifilm) and quantification of relative labeling efficiency (ImageJ). At the end of the time course (2 h) the reaction was quenched by addition of 2-ME and labeled protein was purified by gel filtration (Superdex 75, GE

Healthcare), concentrated (Amicon Ultra 10 kDa MW cutoff), and quantified by absorbance to determine the labeling efficiency. These experiments revealed that the single-cysteine variant SecYEG(298) was labeled much more rapidly than SecYEG(148) under identical reaction conditions (*SI Appendix,* Fig. S1*A*). This kinetic selectivity allowed us to label the double-cysteine variant SecYEG(148/298) for 90 s to obtain preferential labeling at position 298 (50%), and very little labeling at position 148 (8%). After purification of Cy3-SecYEG by gel filtration (Sephadex G-25, GE Healthcare), remaining cysteines were reacted with Atto647N-maleimide (Atto-Tec) for 2 h using a 75 µM Cy3-SecYEG and 375 µM fluorescent dye. Double-labeled SecYEG was purified by gel filtration as above prior to nanodisc assembly. The final material contained 0.5 molecules of Cy3 (donor) and 1.3 molecules of Atto647N (acceptor) per SecYEG, in line with labeling time courses of single-cysteine variants. This approach ensured that particles with a donor at position 298 and an acceptor at 148 were in large excess (12:1) over inversely labeled particles. Atto488/Atto647N labeled SecYEG(148/298) was prepared similarly. Protein and dye concentrations were determined by absorbance, using the following extinction coefficients: SecYEG: $\varepsilon_{280}$ =71 mM$^{-1}$cm$^{-1}$; Atto488: $\varepsilon_{501}$ =90 mM$^{-1}$cm$^{-1}$, Cy3: $\varepsilon_{548}$ =162 mM$^{-1}$cm$^{-1}$, Atto647N: $\varepsilon_{644}$ =150 mM$^{-1}$cm$^{-1}$.

**Nanodisc assembly.** Nanodiscs containing SecYEG were prepared from purified SecYEG, biotin-coupled MSP1D1 protein, and total *E. coli* phospholipids (Avanti Polar Lipids) and isolated by size-exclusion chromatography on a Superdex 200 column according to published protocols (2). Nanodiscs containing SecYEG and YidC were prepared as above with addition of YidC at a concentration equal to that of SecYEG. To confirm the presence of YidC and SecYEG in nanodiscs, the nanodisc band was excised from native-PAGE and run on SDS-PAGE to reveal the presence of SecYEG, MSP and YidC proteins in a ratio of approximately 1:1 (*SI Appendix,* Fig. S9). Assembly of YidC and SecY in the same nanodisc was confirmed by crosslinking and western blotting as follows. SecYEG (100 µM) was first functionalized by reaction with the UV-inducible crosslinker sulfo-NHS-diazirine (1 mM, Sigma) in buffer D for 2 h on ice before quenching with 100 mM Tris, pH7.5. Excess crosslinker was then removed by desalting with a PD-10 column. The functionalized SecYEG was then assembled into nanodiscs with YidC and purified prior to crosslinking induced by UV irradiation (365 nm) for 10 min at 4°C. All procedures prior to UV irradiation were done under subdued light. Crosslinking of functionalized SecYEG to YidC was confirmed by Western blotting using an anti-YidC antibody (gift from H. G. Koch, University of Freiburg, Germany) (*SI Appendix,* Fig. S9).

**RNC preparation.** Ribosomes from *E. coli* MRE600, initiation factors (IF1, IF2, IF3), elongation factors (EF-Tu, EF-Ts, EF-G), and initiator tRNA (f[$^{3}$H]Met-tRNA$^{fMet}$) were prepared according to standard protocols (5-9). Total tRNA from *E. coli* was purchased from Roche and aminoacylated as described elsewhere (10) with inclusion of [$^{14}$C]Leu. mRNAs were prepared by *in vitro* transcription and purified according to a published protocol (11). 70S initiation complexes were prepared by incubating 70S ribosomes (1 µM), 1.5 µM of each initiation factor, excess mRNA (variable, saturation verified by titration), 2.5 µM f[$^{3}$H]Met-tRNA$^{fMet}$, and 1 mM GTP at 37°C for 1 h in buffer A. Initiation efficiency was determined from the absorbance at 260 nm and by nitrocellulose filtration and scintillation counting of [$^{3}$H]Met.

RNCs were prepared by *in-vitro* translation in HiFi buffer (50 mM Tris, pH 7.5, 70 mM $NH_4Cl$, 30 mM KCl, 3.5 mM $MgCl_2$, 8 mM putrescine, 0.5 mM spermidine) using 0.125 µM 70S initiation complex, 15 µM EF-Tu, 0.25 µM EF-G, 0.02 µM EF-Ts, total aminoacyl-tRNA containing 6.75 µM [$^{14}$C]Leu-tRNA$^{Leu}$, 1 mM GTP, 3 mM phosphoenolpyruvate, and 10 µg/ml pyruvate kinase at 37°C for 1 h. RNCs were purified by centrifugation through a sucrose cushion (400 µl, HiFi buffer containing 1.1M sucrose) in a TLS-55 rotor (Beckman) at 55,000 rpm for 2 h. Pellets containing RNCs were dissolved in buffer A, flash-frozen and stored at -80°C until use. Translation efficiencies were better than 70%, as determined by scintillation counting of [$^{14}$C]Leu in the nascent chain, and the absorbance at 260 nm.

**Assay for functional activity of double-labeled SecYEG.** The activity of nanodisc-embedded SecYEG labeled with Cy3 and Atto647N was confirmed by testing the ability to protect radiolabeled nascent chain in an RNC from digestion by proteinase K (PK). Purified LepB75-RNC (100 nM) carrying [$^{3}$H]Met at the N-terminus was incubated with or without SecYEG (500 nM) for 10 min at room temperature in buffer A prior to addition of 1 mg/ml PK and 1 mM $CaCl_2$ for PK activation (final concentrations). Digestion by PK was carried out for 60 s at 37°C and was quenched by addition of KOH (500 mM). Nascent chain was released from the RNC by workup at 37°C for 30 min, followed by TCA precipitation (addition of 7.5 volumes of cold 10% TCA and 30 min incubation on ice). Precipitated peptides were collected on 0.45 µm nitrocellulose (Sartorius), washed first with 10 ml of cold 5% TCA and then with 5 ml cold 30% isopropanol before scintillation counting. Protection from PK digestion was quantified as the amount of precipitated [$^{3}$H]Met, and is presented relative to protection by wild-type (unlabeled) SecYEG (*SI Appendix,* Fig. S1*B*).

**Single-molecule FRET experiments on TIRF.** All smFRET experiments were performed in buffer A with the addition of bovine serum albumin (BSA; 1 mg $ml^{-1}$), protochatechuic acid (5 mM), Trolox (6-hydroxy-2,5,7,8-tetramethylchromane-2-carboxylic acid; 2 mM), protochatechuate 3,4-dioxygenase (50 nM) and methyl viologen (1 mM). For experiments with ligands, RNCs (100 nM), 70S ribosomes (100 nM) or FtsY (2 µM) and GDPNP (0.5 mM), were added to the imaging buffer.

Nanodiscs containing biotin-linked MSP1D1 protein were immobilized on Biotin/PEG functionalized cover slips according to published protocols (12). Cover slips were additionally washed with 1 mg $ml^{-1}$ BSA in buffer A prior to application of imaging buffer. TIRF imaging was performed on an IX 81 inverted microscope (Olympus) using 561 nm solid-state laser excitation (25 mW) as previously described (13). Images were separated into donor and acceptor channels using a beam splitter, and recorded on a CCD camera (CCD-C9100-13; Hamamatsu) at a rate of 33 frames/s for a total of 1000 frames. A minimum of 120 movies were collected for each experiment, and the number of traces used for analysis (250-500) is indicated along with the corresponding FRET histograms (*SI Appendix,* Fig. S6).

**TIRF data analysis.** Fluorescence time courses for donor (Cy3) and acceptor (Atto647N) were extracted as previously described (13). Briefly, a semi-automated algorithm was used to select anti-correlated fluorescence traces (correlation coefficient < -0.4) characteristic of FRET. Time traces for further analysis were selected from the data set by choosing only traces that contained single photobleaching steps for acceptor, and then donor. The bleed-through of donor signal into the acceptor channel was measured for each SecYEG construct using a donor-only labeled sample and was applied as a correction to acceptor fluorescence. Each trajectory was then smoothed once over three data points. FRET efficiency was corrected for relative quantum yields and detection efficiencies of donor and acceptor. This correction was determined empirically from each data set and was found to be consistently 1.6 (acceptor/donor). Corrected FRET efficiency was then calculated according to equation 1:

$$FRET = \frac{A-b\cdot D}{(A-b\cdot D)+D\cdot g} \qquad \text{(equation 1)},$$

where *A* is the acceptor fluorescence, *D* is the donor fluorescence, *b* is the bleed-through correction, and *g* is the correction for detection efficiency.

FRET-histograms were fitted to Gaussian distributions using GraphPrism software. Fits to functions containing different numbers of Gaussian components were considered robust if the interdependencies of the fitted parameters were below 0.9, and then compared using Akaike Information Criterion (AIC, *SI Appendix* Fig. S2, Table S1). The vbFRET software package

(http://vbfret.sourceforge.net/) (14) was used for HMM analysis of the FRET data. In order to globally fit traces, all traces from one data set were compiled into a single long FRET trace. Traces were ordered randomly within the combined trace, and traces were separated by Monte Carlo simulation with a FRET efficiency equal to 10, which was reliably identified by the fitting algorithm. The average length of each Monte Carlo simulation was 100 frames, the same average length as in the experimental traces. We have tested the robustness of the method by a) randomizing the order in which traces were stitched together, b) varying the average length of simulation between traces (10, 100, or 1000 steps), and c) varying the imaginary FRET state (FRET = 2 or 10). All approaches produce the same rate constants and state populations reported in *SI Appendix,* Table S2.

Fitting FRET data revealed that vbFRET consistently identifies the most complex HMM as the best-fit model. To identify an optimal model using an unbiased statistical approach we employed Bayes information criterion. After fitting HMMs from 2-20 states using vbFRET, idealized FRET traces were used to compute the log-likelihood of each model, and Bayes Information criterion was computed which weighs goodness of fit against model complexity (*SI Appendix,* Fig. S3). We observed an improvement in fitting with up to ten states, above which overfitting outweighed goodness of fit (*SI Appendix,* Fig. S3*A*). Above nine states, individual FRET states become redundant (i.e., overlap almost completely), indicating that the smFRET data could be adequately described by up to nine states (*SI Appendix,* Fig. S3*B*,*C*). FRET traces from TIRF experiments are shown along with the fitting results from 4-state and 9-state models (*SI Appendix*, Fig. S3*F*,*G*). Some small FRET differences identified by the 9-state HMM (*SI Appendix,* Fig. S3*F*,*G*) may be similar in magnitude to small variations in fluorescence inherent to the experimental conditions (orientation of the nanodisc or local excitation intensity, for example), and argue in favor of a simpler (4-state) model. We note that fits with models containing four states to nine states support a linear kinetic mechanism, which cannot be explained by local experimental variations. In addition, fits with these models reveal transitions between FRET states on similar timescales, in the range of 1-10 $s^{-1}$ (Fig. 3 and *SI Appendix,* Fig. S3*D*,*E*).

Stochastic rate constants were determined by dwell-time analysis of the idealized FRET traces (15). Dwell times were corrected for the finite length of each trace (defined manually at the acceptor photobleaching event), which was manifested in the idealized combined trace by transition to the Monte Carlo simulation. The uncertainty in each stochastic rate constant was defined as the 95% credible interval, and computed using the inverse function of the regularized incomplete beta function as described (15).

All model-free analysis was performed in Matlab. FRET histograms obtained from TIRF experiments were compared using the Kolmogorov-Smirnov (K-S) test to quantify the largest difference in the empirical cumulative distribution functions obtained from two experiments (16). For significance testing, 1000 simulated data sets were constructed for each experiment by empirical Monte Carlo simulation. Similarly, the range of the FRET peak for each experiment was determined by identifying the peak FRET value in each of the 1000 simulated data sets. Each simulated data set was comprised of N simulated time traces, where N is the number of traces in the corresponding experimental data set. The length of each time trace was sampled randomly from an exponential distribution with mean equal to 100 steps (same mean as experimentally obtained traces), and only traces longer than 12 steps were permitted. For each time trace, the starting fluorescence was sampled randomly from the kernel density estimate of the experimentally obtained FRET histogram. For each step in the simulation, the change in FRET was determined by random sampling from an empirically derived step function. Step functions were obtained by kernel density estimate smoothing of experimentally observed FRET changes for data points originating in the same region of the FRET histogram over a FRET window of 0.03. The simulated data sets were found to adequately capture the stochastic noise of the FRET histograms. To validate this approach, the SecYEG TIRF traces were partitioned randomly into two test sets and compared by K-S testing. Each test set was used to derive 1000 simulated data sets, and comparison by K-S testing revealed no significant difference between the data ($p = 0.16$). For K-S testing, p-values were determined by comparing the test statistics computed from two experimental data sets to the test statistics computed from the experimental data and each of the corresponding simulated data sets (2000 values for each K-S test). Peak ranges were defined for each experiment by the minimum and maximum peak values observed in histograms of the corresponding simulated data sets. For histogram peaks, p-values were determined by the probability that a simulation based on the experimental data would have a peak value larger than that observed from a simulation based on the SecYEG-alone data.

**Pulsed-interleaved excitation FRET experiments.** PIE-FRET was performed using the MicroTime 200 system (PicoQuant, Berlin, Germany), which is based on a modified Olympus IX 73 confocal microscope and equipped with a water objective lens with 60x magnification and 1.2 numerical aperture (Olympus UPlanSApo). Alternating 485 nm and 640 nm laser excitation (PIE mode) was carried out at 20 MHz and focused through the objective into the sample. The laser power was set to 80 and 12 µW, respectively. Fluorescence signals were collected using the same objective (epifluorescence configuration) and separated from the excitation light by a

dichroic mirror. From there, the collected fluorescence light was focused through a 50-µm pinhole to eliminate fluorescence coming from axial positions away from the focal plane (confocal detection). A beam splitter (T635 lpxr) was used to split the fluorescence signal into donor and acceptor channels, which were each filtered and focused onto single-photon avalanche photodiodes (SPADs). Fluorescence-labeled nanodiscs were measured in buffer A with 1 mg $ml^{-1}$ BSA, and the concentration was adjusted to yield an average of less than 0.1 molecules within the confocal detection volume. Each measurement was performed at 22°C for 15 min using freshly diluted sample. A total of 21 measurements (5.25 hours measurement time) were compiled for data analysis.

**PIE-FRET data analysis.** PIE-FRET data were analyzed using PIE analysis with Matlab (PAM) software(17). Bursts were identified using a sliding time window algorithm with a minimum of 100 photons per burst, a 500-µs time window, and 5 photons per time window. Burstwise lifetime analysis was performed using bi-exponential reconvolution fitting with a measured instrument response function (IRF) (Ludox colloidal silica, Sigma), and background measurement (unlabeled SecYEG-nanodiscs). Corrections for detector crosstalk and direct excitation (determined from the donor-only and acceptor-only populations) were found to be 1.8% and 2.3%, respectively. After removing the donor-only population (about 10% of all particles), the population with one donor and one acceptor (stoichiometry = $n_D/(n_D+n_A)$ = 0.5), was selected by fitting a three-Gaussian function to a 2D plot of donor count rate *vs*. stoichiometry. This procedure separated the acceptor-only population (about 10% of all particles), and a population of particles with low stoichiometry (about 25%) from the particles with one donor and one acceptor (about 33% of the total data set). The latter population comprised 17,000 molecules and was used for further analysis. This data set was then corrected for relative quantum yields ($\Phi$) and detection efficiencies ($g$) of donor and acceptor fluorophores by fitting a plot of donor lifetime vs. intensity-based FRET efficiency to the static FRET line (Fig. S4*A*). The resulting correction factor was $\gamma = (\Phi_A g_A)/(\Phi_D g_D) = 1.64$.

Time-window analysis was performed by dividing each burst into time windows with a length of 3, 1, 0.3, or 0.1 ms and computing the resulting FRET histogram for time windows with more than 25 photons ($D_{ex}D_{em}+D_{ex}A_{em}$) (Fig. 4*A*). The broad shape of the FRET histogram observed without time window analysis persisted at all time windows investigated. If dynamic transitions on the millisecond timescale were responsible for FRET broadening, time window analysis would reveal two or more narrow FRET populations.

Autocorrelation of acceptor fluorescence was computed over ten equal time intervals during a 15 min measurement (black, mean and standard deviation), and the curve was fitted to a function

with one diffusion time ($\tau_D$ = 0.74 ms) and one triplet state ($\tau_{Trip}$ = 35 µs, amplitude = 7.5%). The following equation was used for fitting the autocorrelation data:

$$G(t) = \frac{1}{\sqrt{8N}}\left[1 + Ae^{-t/\tau_{\text{Trip}}}\right]\left[\frac{1}{1+\tau/\tau_D}\right]\left[\frac{\tau/\tau_D}{\sqrt{1+1/\alpha^2}}\right] \quad \text{(equation 2)},$$

where N is the average number of molecules in the focal volume, A is the amplitude of the triplet state, $\tau_{Trip}$ is the lifetime of the triplet state, $\tau_D$ is the diffusion time, and α is the ratio of axial to radial radii of the focal volume. Fitting four independent measurements yielded $\tau_D$ = 0.74±0.04 ms, $\tau_{Trip}$ = 35±7 µs, and a triplet state amplitude of 7.5±0.1% (mean ± standard deviation). The small contribution of this triplet state (<10%) indicates that acceptor photophysics cannot explain the large standard deviation computed in BVA.

Burst Variance Analysis (BVA) was performed using PAM software(17) in order to investigate the possibility of dynamic changes at the lateral gate of SecYEG, while diffusing through the confocal volume. BVA involves calculating the variability of FRET efficiencies measured for each single molecule as it diffuses through the confocal volume(18) . First, single molecules (bursts) were sorted based on the FRET efficiency calculated over the entire diffusion time, and placed in one of 20 evenly spaced FRET bins. The bursts in each bin were then divided into continuous windows containing an equal number of photons (n=5), and the standard deviation of FRET, σ(FRET), is computed for all windows and all molecules in the bin. The σ(FRET) is then plotted for each molecule (contour in Fig. 4*B*) and for all windows and all molecules in each bin (blue symbols in Fig. 4*B*) using a minimum of 100 molecules per bin. The expected (theoretical) dependence of σ(FRET) on FRET, assuming a single (static) FRET state, is curved with minima at 0 and 1 FRET efficiency and a maximum at moderate FRET efficiency, and describes the stochastic variability in FRET values measured from a small number of photons (black line in Fig. 4*B*). The 99.9% confidence intervals (gray shaded area in Fig. 4*B*) were computed by Monte Carlo simulation.

**Accessible volume simulations.** Accessible volume simulations of Cy3 (donor) and Atto647N (acceptor) fluorophores were performed using the FRET Positional Screening (FPS) software (19). Models for the closed and open conformation of SecYEG were obtained from the protein data bank (PDBIDs 3J45 and 3J00, respectively). In order to prevent the dyes from sampling the space occupied by phospholipids in the nanodisc, the nanodisc model (lipids and MSP protein) from 3J00.pdb were included in both closed and open conformations. For the closed conformation, the relative position of the nanodisc was determined by superimposition of SecY structures from both pdb files. To model the experimental dye-attachment positions, residues 148

and 298 were replaced with cysteine sidechains using the mutagenesis plugin in Pymol (Schroedinger, LLC), and the sulfur atoms were selected as the attachment positions. The fluorophores were modeled as 3-dimensoinal ellipsoids attached with flexible linkers using the following parameters (in Å): Cy3:$L_{link}$=21, $w_{link}$=4.5, $R_1$=6.8, $R_2$=3.0, $R_3$=1.5, Atto647N: $L_{link}$=21, $w_{link}$=4.5, $R_1$=7.15, $R_2$=4.5, $R_3$=1.5.

## Supporting Figures

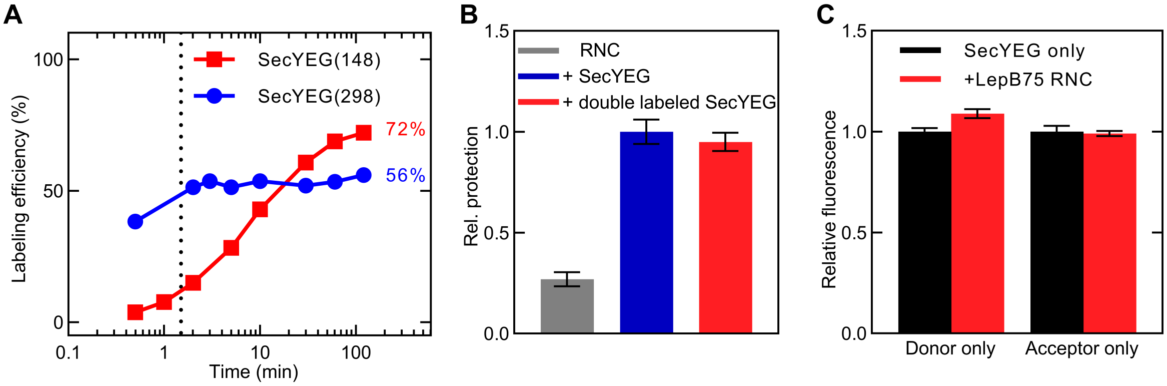


**Figure S1. Fluorescence labeling of SecYEG.** (*A*) Kinetic selectivity of donor (Cy3) labeling was measured using single-cysteine variants of SecYEG as described in SI Methods. The vertical dotted line indicates 90 s, the incubation time used for selective labeling of position 298 when double-labeled SecYEG (148/298) was generated. (*B*) Activity of Cy3/Atto647N-labeled SecYEG was assayed by testing the ability to protect radiolabeled LepB75-RNC nascent chain from PK digestion. The protected peptide was TCA precipitated and $^{3}$H-methionine quantified by scintillation counting. Radioactivity is plotted relative to protection by wild-type (unlabeled) SecYEG. Bars represent averages ± standard deviation (n=4). (*C*) Fluorescence of donor and acceptor labels were measured in bulk without (SecYEG only) and with addition of LepB75-RNC. Donor-only fluorescence was measured using Cy3-labeled SecYEG in nanodiscs ($\lambda_{ex}$ = 510 nm, $\lambda_{em}$ = 566 nm) and acceptor only fluorescence was measured via direct acceptor excitation of Cy3/Atto647N labeled SecYEG in nanodiscs ($\lambda_{ex}$ = 610 nm, $\lambda_{em}$ = 660 nm). The concentration of SecYEG-nanodisc was 50 nM and LepB75-RNC was 100 nM when present. Measurements indicate a small (< 10%) donor increase upon LepB75-RNC binding and no change in acceptor fluorescence. Bars represent averages ± standard deviation (n=3).

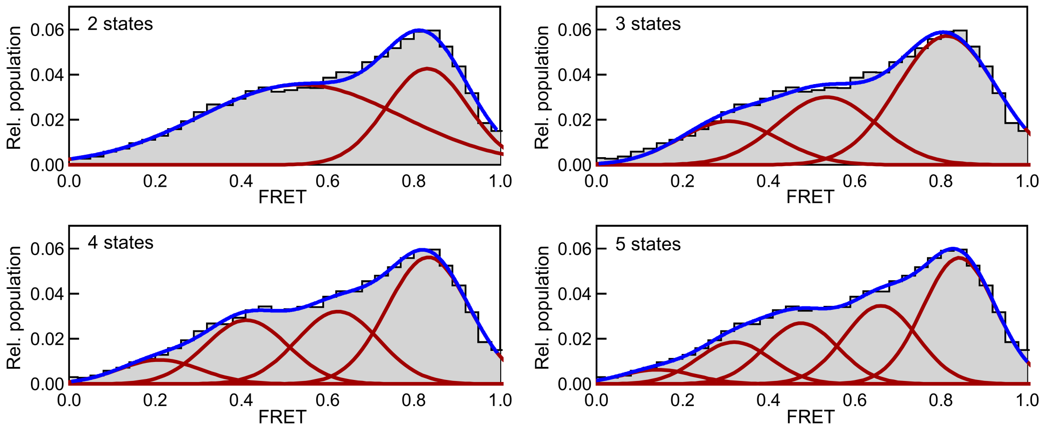


**Figure S2. Gaussian fitting of smFRET histograms.** Single-molecule FRET histograms from nanodisc-reconstituted SecYEG complexes (gray) were fitted with different models. Blue curves show the cumulative distribution containing increasing numbers of Gaussian functions (red) used for fitting the histograms. The broadness of the FRET distribution suggests that a number of different models would fit the data reasonably well. Consistent with this expectation, fitting functions with two, three, or four Gaussian components each provided reasonable fits to the FRET histogram with $R^2$ values in the range of 0.98-0.99 (Table S1). With three or more Gaussian components, however, the components were not well separated, and some fitted parameters had very high interdependencies (>99 %), indicative of overfitting. This issue was resolved by linking the standard deviations in the Gaussian components, thus reducing the number of fitted parameters in the 3-Gaussian and 4-Gaussian functions to 7 and 9, respectively. The resulting fits were still of high quality ($R^2$ = 0.98 and 0.99 for 3-Gaussian and 4-Gaussian, respectively), and all parameters had interdependencies below 90%. Fitting to a 5-Gaussian function produced parameters with high interdependencies (>96%), even after linking the standard deviations across all five Gaussian components. All fits with small interdependencies in the fitted parameters were compared using Akaike Information Criteria (AIC)(20). This metric is proportional to the goodness of fit, but includes a penalty for each fitted parameter, thus balancing underfitting and overfitting. Comparison of the AIC values reveals that the best-fit models are 4-Gaussian>2-Gaussian<3-Gaussian, with the 4-Gaussian model preferred over the 2-Gaussian with a likelihood of 99%.

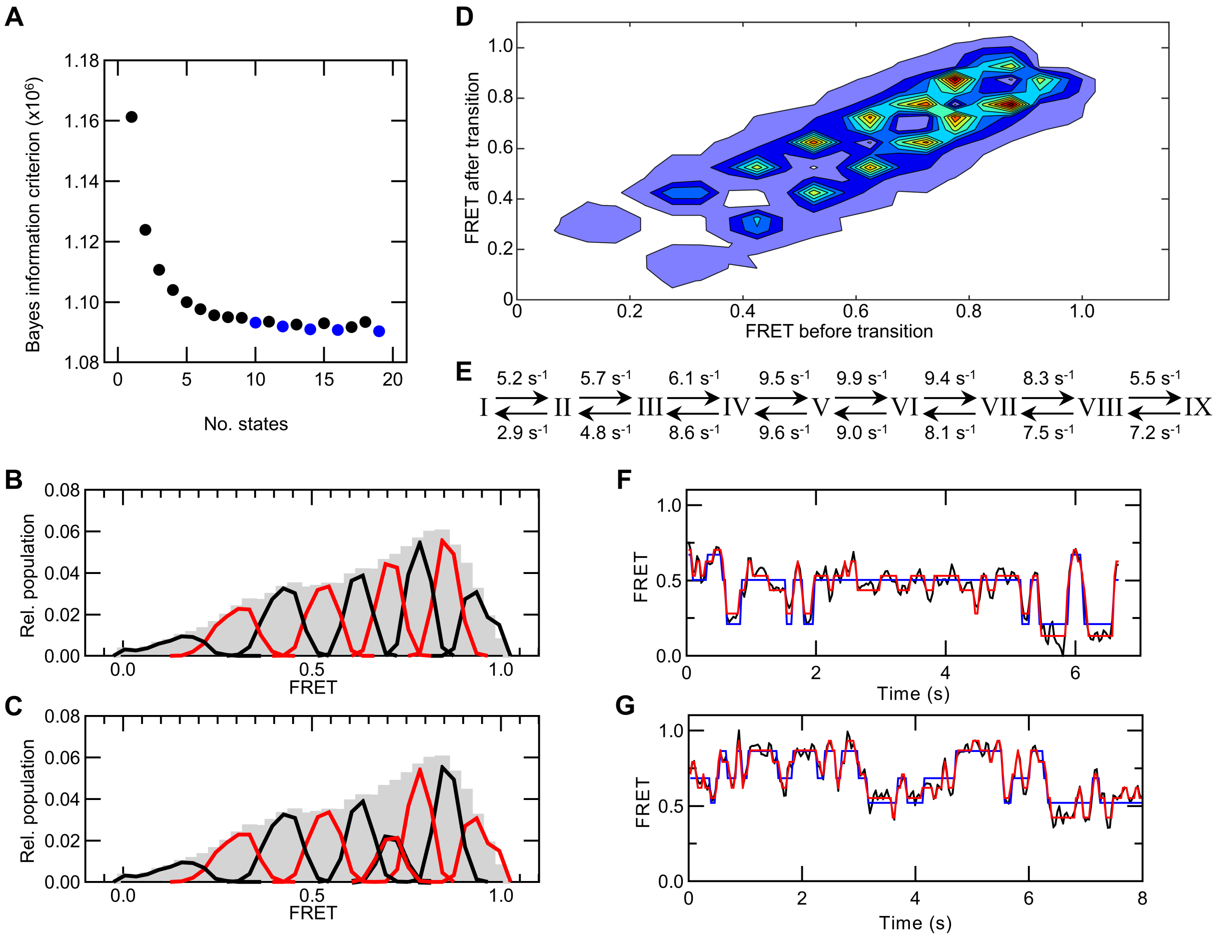


**Figure S3. Comparison of fitted HMM models with 1-19 states.** (*A*) The Bayes Information Criterion from HMM fitting was computed for each fitted model. Local minima are indicated by blue symbols. The FRET histogram obtained from SecYEG (gray) is plotted behind FRET values binned according to the states identified from a nine-state (*B*) or a ten-state (*C*) HMM. Different states in panels (*B*) and (*C*) are colored red and black for clarity. Bayes Information Criterion (BIC) was defined as BIC=$k$ ln($n$)-2ln($L$) (20), where $k$ is the number of fitted parameters, $n$ is the number of data (time) points, and $L$ is the likelihood computed for the best-fit model. As the likelihood (goodness of fit) increases, the BIC decreases, while increasing the number of fitted parameters introduces a penalty. A minimum BIC, therefore, indicates the best balance between goodness of fit and number of states. (*D*) Transition density plot indicating the average FRET before and after each transition identified by nine-state HMM analysis. (*E*) Kinetic mechanism of lateral gate opening derived from nine-state HMM analysis of smFRET data. (*F-G*) Representative FRET traces from TIRF microscopy (black) with idealized traces from 4-state (blue) and 9-state (red) Hidden Markov Models.

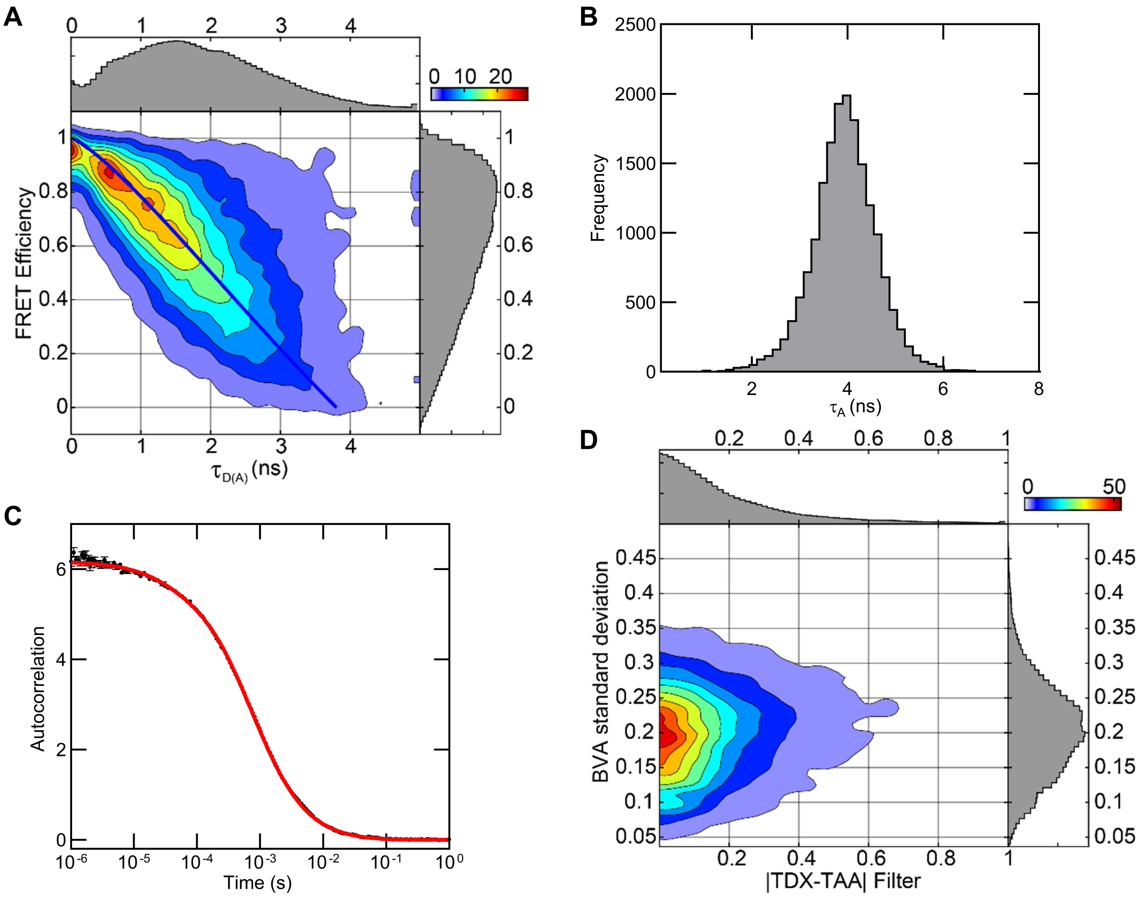


**Figure S4. PIE-FRET analysis of freely diffusing nanodisc-reconstituted SecYEG.** (*A*) Single-molecule lifetime analysis showing the negative correlation between intensity-based FRET efficiency (y-axis) and donor lifetime in the presence of acceptor (x-axis). The blue line indicates the theoretically predicted dependence, and the contours represent the number single molecules colored from white to red in increasing number according the color legend. (*B*) Acceptor lifetimes upon acceptor excitation are unimodal with $<\tau_A>$ = 3.9 ns indicating a single state of the acceptor. (*C*) Autocorrelation of acceptor fluorescence (black, mean and standard deviation), and the curve was fitted to a function with one diffusion time ($\tau_D$ = 0.74 ms) and one triplet state ($\tau_{Trip}$ = 35 µs, amplitude = 7.5%). (*D*) In order to address the possibility that FRET broadening results from photobleaching of one fluorophore before the other, we compared the difference in burst-averaged photon arrival times after donor (TDX) and acceptor (TAA) excitation (|TDX-TAA|)(21). This quantity represent the time difference between the average photon arrival time and the first photon in each burst (in ms). Photobleaching would cause one fluorophore to stop emitting before the other, and produce large values of |TDX-TAA|. The vast majority of burst in the experiment have |TDX-TAA| values below 0.7 ms, which is the recommended cutoff to remove photobleaching(21). Lack of correlation between |TDX-TAA| and with σ(FRET) calculated by BVA

analysis (BVA standard deviation) is a clear indication that FRET broadening is not due to photobleaching.

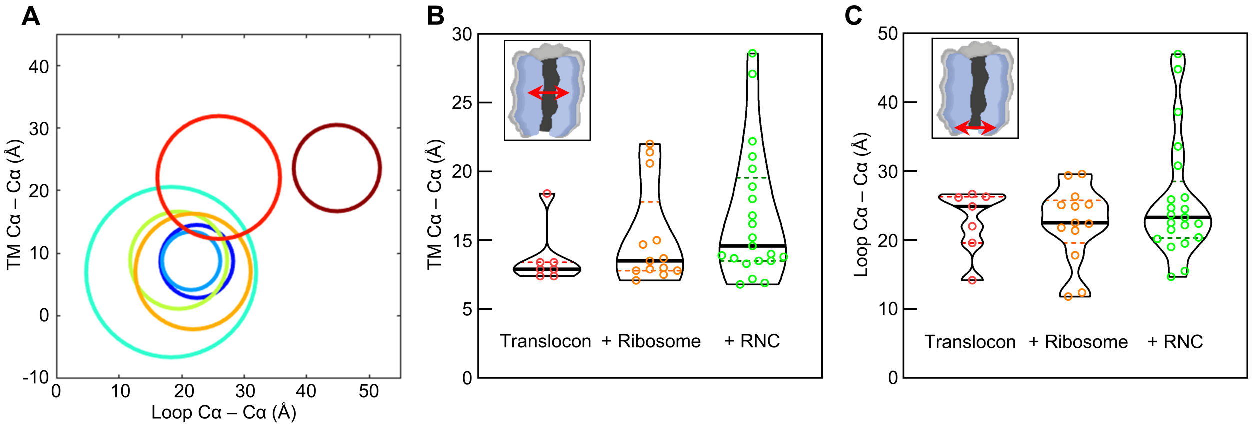


**Figure S5.** Lateral gate opening measured in available structures. (*A*) Distances between alpha carbons in SecY loops (residues L148 and T298) are plotted versus the corresponding distances between alpha carbons of the lateral gate TMs (residues S87 and F286, previously used for PET-based fluorescence measurements (2)) for each *E. coli* translocon structure in the PDB. The radius of each circle is equivalent to the reported resolution (in Å), and each structure is plotted in a different color for clarity. Lateral gate opening is characterized by a positive correlation between TM-TM distance and loop-loop distance. The limited resolution of available structures, however, prevents a more detailed comparison. In particular, it is unclear if structures with a closed lateral gate have different loop conformations. (*B*) Distances between Cα atoms in the lateral gate (same as (*A*)) of the translocon are shown as violin plots for all structures in the PDB (including non-*E. coli* structures). Inset shows schematic of the translocon with the location of the measured distance by a red arrow. (*C*) Same as (*B*) but for loop residues where FRET labels were positioned in this study.

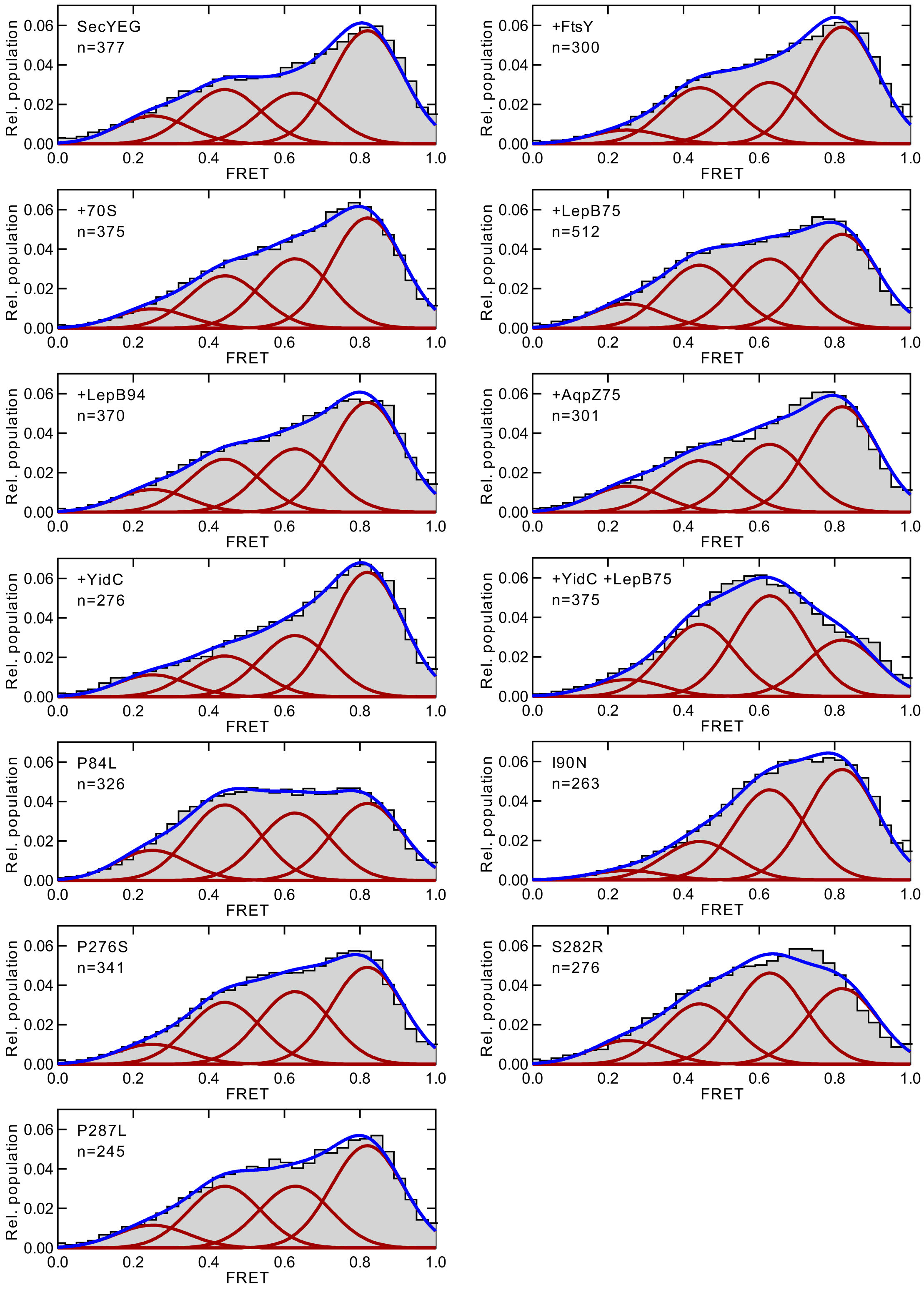


**Figure S6. Four-state Gaussian analysis of the lateral gate of SecYEG.** Single-molecule FRET histograms from nanodisc-reconstituted SecYEG complexes were obtained in the presence of different ligands (FtsY, YidC, various RNCs) or for SecYEG variants. The blue curve

indicates the cumulative distribution containing four Gaussian functions (red) that was fitted to each histogram. The mean and standard deviation for each FRET state was fitted globally between all data sets. The number of particles analyzed is indicated (n).

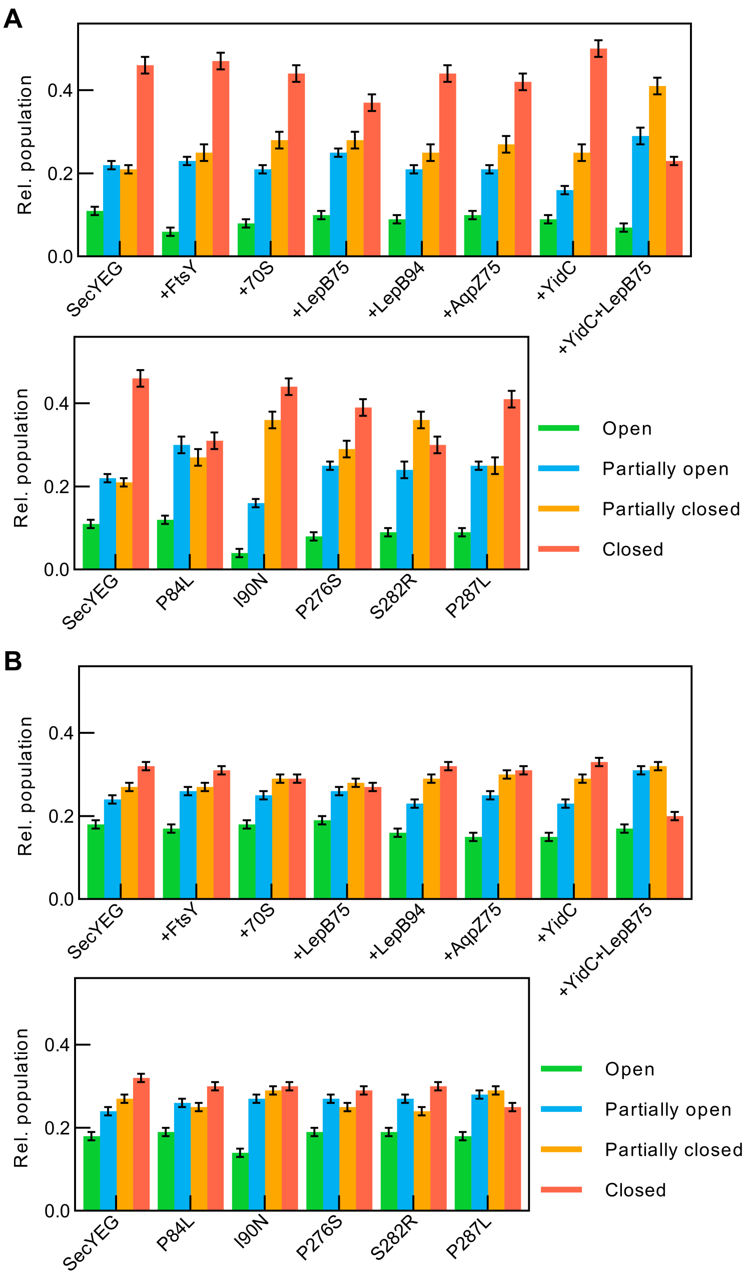


**Figure S7. Quantification of FRET populations from four-state Gaussian and HMM analysis.** (*A*) The populations of each FRET state calculated from Gaussian fitting (Fig. S6) are shown as bars. The FRET states are labeled from low FRET to high FRET as follows: open (green), partially open (blue), partially closed (orange), and closed (red). (*B*) Same as (*A*) for HMM analysis.

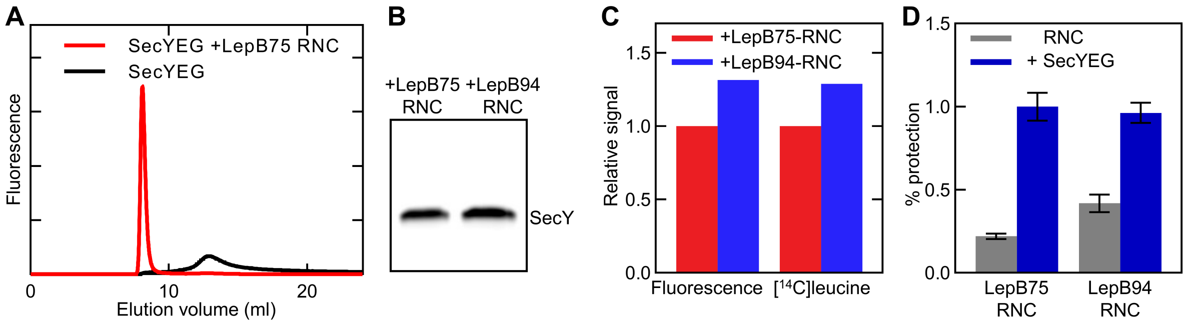


**Figure S8. Characterization of the SecYEG-RNC interactions.** (*A*) Quantification of translocon binding to the RNC. Fluorescence-labeled Cy3/Atto647N SecYEG-nanodiscs were subjected to gel filtration on Superdex 200 (10/300 GL, GE Healthcare) in buffer A with (red) or without (black) prior incubation with LepB75-RNC (+LepB75-RNC or SecYEG only, respectively). Elution of the translocon was monitored using a fluorescence detector via direct acceptor excitation ($\lambda_{ex}$ = 635 nm, $\lambda_{em}$ = 670 nm) and chromatograms were normalized based on total fluorescence. In the presence of RNC, translocons are found exclusively in the complex with SRP. (*B*) Isolation of the RNC-translocon complex. Pull-down of His-tagged Cy3/Atto647N-labeled SecYEG-nanodiscs with LepB75-RNC or LepB94-RNC. Fluorescence-labeled SecYEG-nanodiscs were incubated with LepB75-RNC or LepB94-RNC, prior to 1 h binding to Ni-NTA resin in buffer A, washing with buffer A + 50 mM imidazole, and elution in buffer A + 500 mM imidazole. SecYEG-nanodiscs in the elution fraction were separated on SDS-PAGE and (*C*) quantified by fluorescence scanning of the Atto647N fluorophore (Amersham Typhoon RGB, Cytiva). Nascent chains in the RNCs co-eluting with SecYEG-nanodiscs were quantified by [$^{14}$C] scintillation counting. Quantification is plotted in bars relative to the sample with LepB75-RNC, and indicates that LepB75-RNC and LepB94-RNC interact with FRET-labeled SecYEG-nanodiscs to the same extent, as the amount of nascent chain scales directly with the amount of translocon in the pull-down. (*D*) Protease protection of LepB75-RNC and LepB-94-RNC were assayed in the presence and absence of SecYEG-nanodiscs upon addition of proteinase K. Protected peptides were TCA precipitated and $^{3}$H-methionine quantified by filtration and scintillation counting. Radioactivity is plotted relative to total input. Bars represent averages with standard deviation (n=4). The data presented for LepB75-RNC is the same as in Fig. *S1B*.

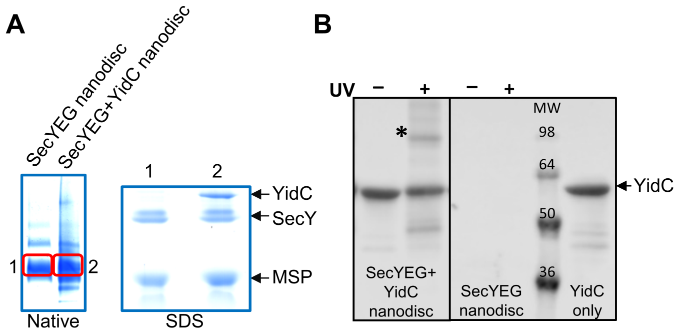


**Figure S9. Characterization of SecYEG-YidC nanodiscs**. (*A*) Analysis of YidC incorporation into nanodiscs. Left panel, nanodiscs were isolated from clear-native PAGE by excising bands as indicated by red frames. Right panel, SDS-PAGE analysis of nanodiscs isolated by native PAGE. Bands in SDS-PAGE corresponding to the nanodisc protein MSP, SecY, and YidC are indicated. The YidC is clearly present in YidC-SecYEG nanodiscs, in approximately 1:1 stoichiometry with SecY. (*B*) YidC and translocon are incorporated into the same nanodisc. Left panel, crosslinking between SecY and YidC detected in SecYEG-YidC nanodiscs via anti-YidC western blot. SecYEG was functionalized with a UV-activated crosslinker (Sulfo-SDA) prior to assembly of SecYEG-YidC nanodiscs, as described (*SI Appendix*, Methods). The appearance of a crosslinked SecYEG-YidC adduct after UV treatment (*) was observed in an anti-YidC western blot, indicating that SecYEG and YidC are in the same nanodisc. Right panel, controls for crosslinking specificity.

**Table S1. Summary of Gaussian fitting**.

| | Model | | | | | |
|---|---|---|---|---|---|---|
| | Sum of 2 Gaussians | Sum of 3 Gaussians (same σ)[a] | Sum of 3 Gaussians | Sum of 4 Gaussians (same σ)[a] | Sum of 4 Gaussians | Sum of 5 Gaussians (same σ)[a] |
| No. of fitted parameters | 6 | 7 | 9 | 9 | 12 | 11 |
| $R^2$ | 0.9847 | 0.9785 | 0.9936 | 0.9911 | 0.9938 | 0.9939 |
| Maximum Interdependency[b] | 0.9365 | 0.8574 | 0.9964 | 0.8894 | 0.9994 | 0.9711 |
| AIC[c] | -423.2 | -407.8 | -443.8 | -432 | -431.5 | -436.9 |

[a] The σ value was locked during fitting to be the same for all Gaussian components.

[b] Interdependency between fitted parameters ranges from 0 to 1. High values (>0.95), indicate that fitted values are almost entirely dependent on the values of other fitted parameters. Here we report the largest interdependency from all pairs of parameters.

[c] Akaike Information Criteria (20) weighs goodness of fit against overfitting, with smaller (more negative) values indicating a better model. See also *SI Appendix,* Fig. S2.

**Table S2. Rate constants ($s^{-1}$) and number of transitions obtained from HMM fitting**

| Rate constant | 4-state | | | 9-state | | |
|---|---|---|---|---|---|---|
| | Rate (1/s) | Number of transitions | Dwell time ≥ 3 frames | Rate (1/s) | Number of transitions | Dwell time ≥ 3 frames |
| $k_{1\to2}$ | 2.2 ± 0.2 | 449 | 394 | 5.2 ± 0.5 | 260 | 178 |
| $k_{2\to1}$ | 1.9 ± 0.1 | 462 | 334 | 2.9 ± 0.2 | 258 | 111 |
| $k_{2\to3}$ | 2.5 ± 0.2 | 633 | 474 | 5.7 ± 0.3 | 518 | 290 |
| $k_{3\to2}$ | 2.4 ± 0.2 | 654 | 465 | 4.8 ± 0.2 | 516 | 247 |
| $k_{3\to4}$ | 2.4 ± 0.2 | 668 | 487 | 6.1 ± 0.3 | 660 | 309 |
| $k_{4\to3}$ | 1.9 ± 0.2 | 667 | 579 | 8.6 ± 0.3 | 682 | 237 |
| $k_{4\to5}$ | | | | 9.5 ± 0.3 | 755 | 256 |
| $k_{5\to4}$ | | | | 9.6 ± 0.3 | 722 | 224 |
| $k_{5\to6}$ | | | | 9.9 ± 0.3 | 745 | 250 |
| $k_{6\to5}$ | | | | 9.0 ± 0.3 | 770 | 243 |
| $k_{6\to7}$ | | | | 9.4 ± 0.3 | 808 | 269 |
| $k_{7\to6}$ | | | | 8,1 ± 0.3 | 831 | 273 |
| $k_{7\to8}$ | | | | 8.3 ± 0.3 | 853 | 324 |
| $k_{8\to7}$ | | | | 7.5 ± 0.3 | 898 | 412 |
| $k_{8\to9}$ | | | | 5.5 ± 0.2 | 658 | 256 |
| $k_{9\to8}$ | | | | 7.2 ± 0.4 | 622 | 368 |

## Supporting References


1. D. J. du Plessis, G. Berrelkamp, N. Nouwen, A. J. Driessen, The lateral gate of SecYEG opens during protein translocation. *J Biol Chem* **284**, 15805-15814 (2009).
2. Y. Ge, A. Draycheva, T. Bornemann, M. V. Rodnina, W. Wintermeyer, Lateral opening of the bacterial translocon on ribosome binding and signal peptide insertion. *Nat Commun* **5**, 5263 (2014).
3. A. Draycheva, T. Bornemann, S. Ryazanov, N. A. Lakomek, W. Wintermeyer, The bacterial SRP receptor, FtsY, is activated on binding to the translocon. *Mol Microbiol* **102**, 152-167 (2016).
4. I. Taufik, A. Kedrov, M. Exterkate, A. J. Driessen, Monitoring the activity of single translocons. *J Mol Biol* **425**, 4145-4153 (2013).
5. M. V. Rodnina, W. Wintermeyer, GTP consumption of elongation factor Tu during translation of heteropolymeric mRNAs. *Proc Natl Acad Sci U S A* **92**, 1945-1949 (1995).
6. M. V. Rodnina *et al.*, Thiostrepton inhibits the turnover but not the GTPase of elongation factor G on the ribosome. *Proc Natl Acad Sci U S A* **96**, 9586-9590 (1999).
7. M. V. Rodnina, Y. P. Semenkov, W. Wintermeyer, Purification of fMet-tRNA(fMet) by fast protein liquid chromatography. *Anal Biochem* **219**, 380-381 (1994).
8. P. Milon *et al.*, Transient kinetics, fluorescence, and FRET in studies of initiation of translation in bacteria. *Methods Enzymol* **430**, 1-30 (2007).
9. H. J. Wieden, K. Gromadski, D. Rodnin, M. V. Rodnina, Mechanism of elongation factor (EF)-Ts-catalyzed nucleotide exchange in EF-Tu. Contribution of contacts at the guanine base. *J Biol Chem* **277**, 6032-6036 (2002).
10. W. Holtkamp *et al.*, Cotranslational protein folding on the ribosome monitored in real time. *Science* **350**, 1104-1107 (2015).
11. E. Mercier, W. Wintermeyer, M. V. Rodnina, Co-translational insertion and topogenesis of bacterial membrane proteins monitored in real time. *EMBO J* **39**, e104054 (2020).
12. S. Adio *et al.*, Dynamics of ribosomes and release factors during translation termination in E. coli. *Elife* **7** (2018).
13. S. Adio *et al.*, Fluctuations between multiple EF-G-induced chimeric tRNA states during translocation on the ribosome. *Nat Commun* **6**, 7442 (2015).
14. J. E. Bronson, J. Fei, J. M. Hofman, R. L. Gonzalez, Jr., C. H. Wiggins, Learning rates and states from biophysical time series: a Bayesian approach to model selection and single-molecule FRET data. *Biophys J* **97**, 3196-3205 (2009).

15. C. D. Kinz-Thompson, N. A. Bailey, R. L. Gonzalez, Jr., Precisely and accurately inferring single-molecule rate constants. *Methods Enzymol* **581**, 187-225 (2016).
16. J. W. Pratt, J. D. Gibbons, "Kolmogorov-Smirnov two-sample tests" in Concepts of Nonparametric Theory. (Springer New York, New York, NY, 1981), 10.1007/978-1-4612-5931-2_7, pp. 318-344.
17. W. Schrimpf, A. Barth, J. Hendrix, D. C. Lamb, PAM: A framework for integrated analysis of imaging, single-molecule, and ensemble fluorescence data. *Biophys J* **114**, 1518-1528 (2018).
18. J. P. Torella, S. J. Holden, Y. Santoso, J. Hohlbein, A. N. Kapanidis, Identifying molecular dynamics in single-molecule FRET experiments with burst variance analysis. *Biophys J* **100**, 1568-1577 (2011).
19. S. Kalinin *et al.*, A toolkit and benchmark study for FRET-restrained high-precision structural modeling. *Nat Methods* **9**, 1218-1225 (2012).
20. D. Posada, T. R. Buckley, Model selection and model averaging in phylogenetics: advantages of akaike information criterion and bayesian approaches over likelihood ratio tests. *Syst Biol* **53**, 793-808 (2004).
21. V. Kudryavtsev *et al.*, Combining MFD and PIE for accurate single-pair Förster resonance energy transfer measurements. *ChemPhysChem* **13**, 1060-1078 (2012).
